# A novel pathway for the photooxidation of catechin in relation to its prooxidative activity

**DOI:** 10.1038/s41598-018-31195-x

**Published:** 2018-08-27

**Authors:** Shunichi Shishido, Rei Miyano, Takuji Nakashima, Hirotaka Matsuo, Masato Iwatsuki, Keisuke Nakamura, Taro Kanno, Hiroshi Egusa, Yoshimi Niwano

**Affiliations:** 1 Tohoku University Graduate School of Dentistry, 4-1 Seiryo, Aoba-ku, Sendai 980-8575 Japan; 2 Kitasato Institute for Life Sciences, Kitasato University, 5-9-1 Shirokane, Minato-ku, Tokyo 108-8641 Japan; 3 Faculty of Nursing, Shumei University, 1-1 Daigaku-cho, Yachiyo, Chiba 276-0003 Japan

## Abstract

In the present study, we evaluated the prooxidative mode of action of photoirradiated (+)-catechin at 400 nm in relation to reactive oxygen species generation and its possible application to disinfection. Photoirradiation of (+)-catechin at a concentration of 1 mg/mL yielded not only hydrogen peroxide (H_2_O_2_) but hydroxyl radical (·OH) in a total amount of approximately 20 μM in 10 min. As a result, photoirradiated catechin killed *Staphylococcus aureus*, and a > 5-log reduction in viable bacteria counts was observed within 20 min. Liquid chromatography-high-resolution-electrospray ionization-mass spectrometry showed that photoirradiation decreased the (+)-catechin peak (molecular formula C_15_H_14_O_6_) whilst it increased two peaks of a substance with the molecular formula C_15_H_12_O_6_ with increasing irradiation time. Nuclear magnetic resonance analysis revealed that the two C_15_H_12_O_6_ peaks were allocated to intramolecular cyclization products that are enantiomers of each other. These results suggest that photoirradiation induces oxidation of (+)-catechin resulting in the reduction of oxygen to generate H_2_O_2_. This H_2_O_2_ is then homolytically cleaved to ·OH, and alongside this process, (+)-catechin is finally converted to two intramolecular cyclization products that are different from the quinone structure of the B ring, as proposed previously for the autoxidation and enzymatic oxidation of catechins.

## Introduction

**Figure 1:**
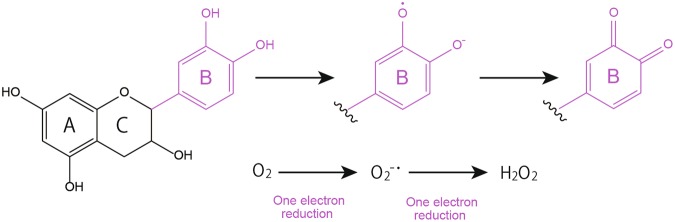
Proposed autoxidation of catechins under certain conditions, such as aerobic heating and pH of >7, coupled with reactive oxygen generation, as reported previously^7,9–11,17^.

Polyphenolic compounds are widely known for their antioxidative activity^1–3^. The antioxidative activity of polyphenolic compounds is mediated by the autoxidation of phenolic hydroxyl groups^4,5^, which in turn leads to radical scavenging activity against free radicals such as the superoxide anion radical (O_2_^−^·) and hydroxyl radical (·OH). Besides antioxidative activity, polyphenols have also the potential to promote oxidation, which is termed prooxidative activity, and this activity is mediated by polyphenol-derived reactive oxygen species (ROS). Since it has been postulated that the prooxidative action of polyphenols is also mediated by the autoxidation of phenolic hydroxyl groups, the antioxidative potential and prooxidative potential of polyphenolic compounds seem to be two sides of the same coin, as reported previously^6^. A typical example of their prooxidative activity is the antibacterial activity of catechins, which is mediated by the hydrogen peroxide (H_2_O_2_) they generate^7^. Besides antibacterial activity, polyphenols’ prooxidative potential has been applied to anti-cancer treatment. It was reported that an important anticancer mechanism of plant polyphenols is intracellular copper mobilization and ROS generation, which is a characteristic feature of the prooxidant properties of polyphenolic compounds, leading to cancer cell death^8^. Taking catechin for instance, as shown in Fig. 1, it has been proposed that the first step of autoxidation is the one-electron oxidation of the B ring of catechins to generate a semiquinone intermediate and a O_2_^−^·, which is in turn further reduced to H_2_O_2_ with the quinone formation of the B ring^7,9–11^.

Novel disinfection techniques, where artificially generated ·OH kills pathogenic fungi and bacteria, have been developed in our laboratory^12,13^, on the basis that ·OH leads to the oxidation of microbial cellular components, such as the cell membrane, nucleic acids, and cell organelles. In the case of the photolysis of H_2_O_2_, as one of the artificial ·OH generation systems, the bactericidal effect depended on the photoirradiation time, indicating that bacterial death was dependent on the ·OH yield^13^. This idea tempted us to combine the prooxidative activity of polyphenols and the photolysis of H_2_O_2_^14–16^. That is, exposing an aqueous solution of polyphenols to blue light leads to the generation of H_2_O_2_, which is in turn homolytically cleaved to ·OH. The resultant ·OH causes oxidative damage leading to bacterial death. In the case of the prooxidative activity of catechins, H_2_O_2_ generation is strongly dependent on the solution pH; in particular, the H_2_O_2_ generation rate increased with rising pH^7^. However, in the case of photoirradiated polyphenols, the pH was not adjusted, and the pH values of the polyphenol solutions were <7.0^14–16^. Therefore, it is speculated that photoirradiation would induce the photooxidation of such polyphenols, even under acidic conditions. However, no evidence for the photooxidation of polyphenols has yet been reported.

As described above, since the prooxidative activity of catechins has been intensively examined^7,9–11,17^, we chose (+)-catechin as an authentic polyphenol, and the purpose of the present study was to verify the photooxidation of (+)-catechin in relation to ROS generation and bactericidal activity.

## Results

### Colorimetric determination of H_2_O_2_ and electron spin resonance (ESR) analysis of •OH

**Figure 2:**
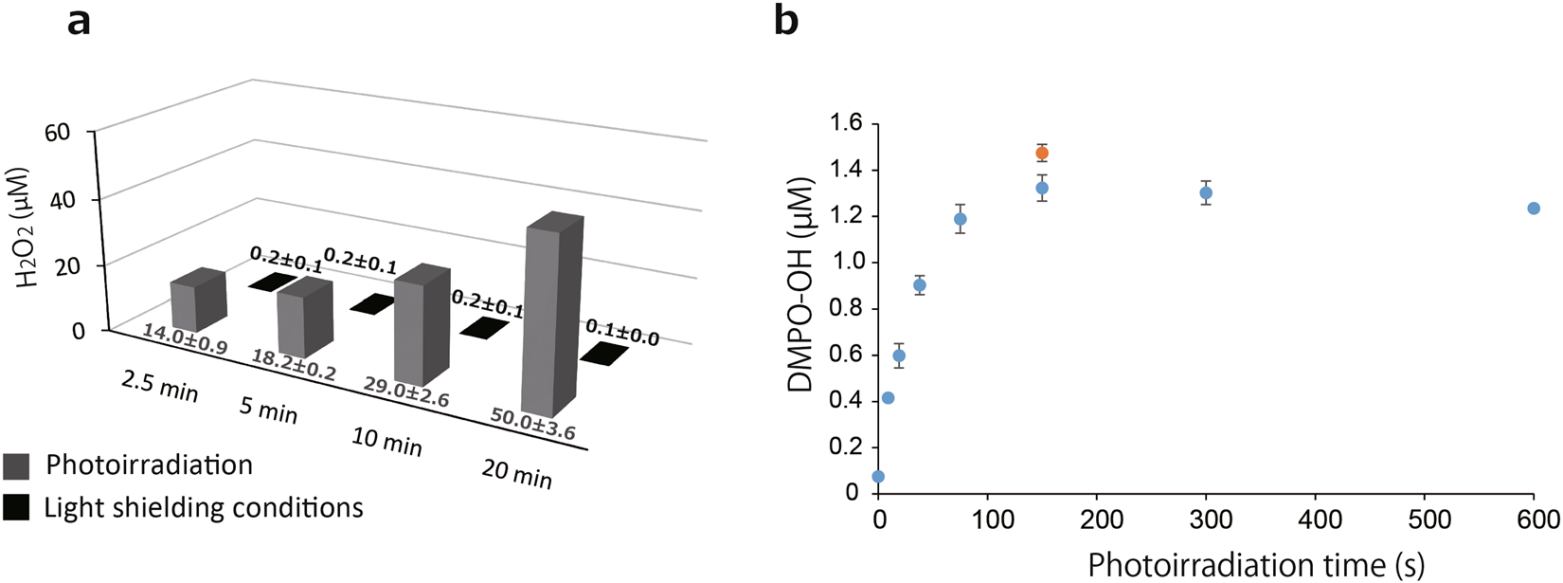
Determination of reactive oxygen species by photoirradiation of (+)-catechin. (**a**) Yield of H_2_O_2_ generated from (+)-catechin (1 mg/mL) during photoirradiation or under light shielding conditions for 2.5–20 min. (**b**) Yield of ·OH (blue colored ●) generated during the photoirradiation of (+)-catechin (1 mg/mL) for 0–600 s, and of ·OH (orange colored ●) generated during a 150 s irradiation of (+)-catechin that was subjected to prior photoirradiation for 600 s without 5,5-dimethyl-1-pyrroline *N*-oxide (DMPO). Each value indicates the mean with the standard deviation (n = 3).

The photoirradiation of (+)-catechin solution (1 mg/mL) at a wavelength of 400 nm generated H_2_O_2_ in an irradiation time-dependent manner (Fig. 2a). By contrast, only trace levels of H_2_O_2_ were found in the (+)-catechin solution kept under light shielding conditions for up to 20 min (Fig. 2a). The average yield of H_2_O_2_ generated in photoirradiated (+)-catechin following a 2.5, 5, 10, and 20 min irradiation time was approximately 14, 18, 29, and 50 µM, respectively. It was confirmed that the generated H_2_O_2_ determined by the method described in the Materials and Methods section was almost completely scavenged by catalase (Fig. S1a). In addition, H_2_O_2_ levels determined by this method were comparable to those by the other method using the Amplex Red/horseradish peroxidase assay^18^ (data not shown).

When a (+)-catechin solution with 300 mM 5,5-dimethyl-1-pyrroline *N*-oxide (DMPO, a spin trapping agent) was similarly photoirradiated, an ESR spectrum of DMPO-OH (a spin adduct of DMPO and ·OH, a_N_ = a_H_ = 1.49 mT) was detected. The amount of DMPO-OH increased with an irradiation time up to 150 s and reached a plateau at least up to 600 s (Fig. 2b). To examine if ·OH was continuously being generated during photoirradiation, the photoirradiated (+)-catechin solution without DMPO for 600 s was further photoirradiated for 150 s in the presence of 300 mM DMPO, and a result showed a similar yield of DMPO-OH (approximately 1.5 μM) to that after 150 s irradiation of the (+)-catechin solution containing 300 mM DMPO without prior irradiation (Fig. 2b).

### Effect of photoirradiation of (+)-catechin on DMPO-OH generated by water sonolysis

**Figure 3:**
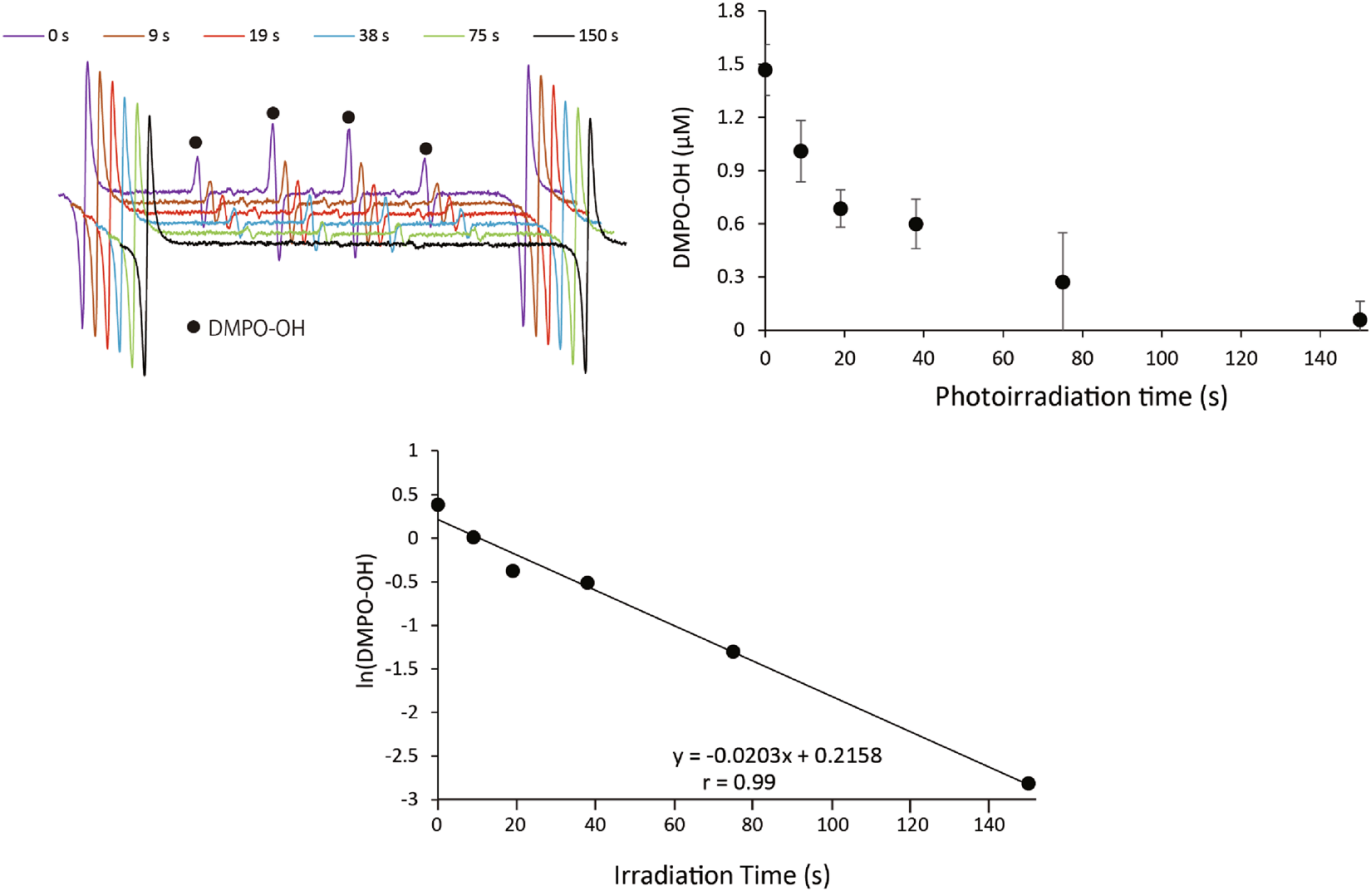
The effect of the photoirradiation of (+)-catechin (1 mg/mL) on DMPO-OH generated during the sonolysis of water. (**a**) ESR spectra. (**b**) Concentration of DMPO-OH. Data are expressed as the mean with standard deviation (n = 3). ESR stands for electron spin resonance.

To examine if DMPO-OH generated by photoirradiated (+)-catechin is degraded or transformed during photoirradiation, DMPO-OH generated through the sonolysis of water was exposed to photoirradiation in the presence of (+)-catechin. When pure water containing 20 mM DMPO was irradiated with ultrasound for 15 s, approximately 3 μM DMPO-OH was yielded, so that the initial DMPO-OH level upon photoirradiation in the presence of 1 mg/mL of (+)-catechin was approximately 1.5 μM, because 250 µL of ultrasound-irradiated pure water was mixed with 250 µL of catechin solution. As shown in Fig. 3a, the ESR signal of DMPO-OH decreased with photoirradiation time in the presence of (+)-catechin. Figure 3b summarizes the decay curve of DMPO-OH obtained through photoirradiation of (+)-catechin, showing that photoirradiation for 150 s resulted in an almost non-detectable level of DMPO-OH. In Fig. 3c, the values of ln(DMPO-OH) are plotted at each time point, showing that the decay reaction of DMPO-OH during the photoirradiation of (+)-catechin is a first-order reaction with the rate constant (*k*_1_) of 0.0203/s and the half-life (*t*_1/2_) of 34.1 s, calculated using the following equation: *t*_1/2_ = (ln2)/*k*_1_. More detailed information is included in the Discussion. In the case of the photoirradiation of (+)-catechin with 10 mM DMPO for 150 s, DMPO-OH was not detected (Fig. S2). In addition, it was confirmed that neither (+)-catechin alone nor photoirradiation alone affected the DMPO-OH level generated through the sonolysis of water (Fig. S2).

### Bactericidal assay

**Figure 4:**
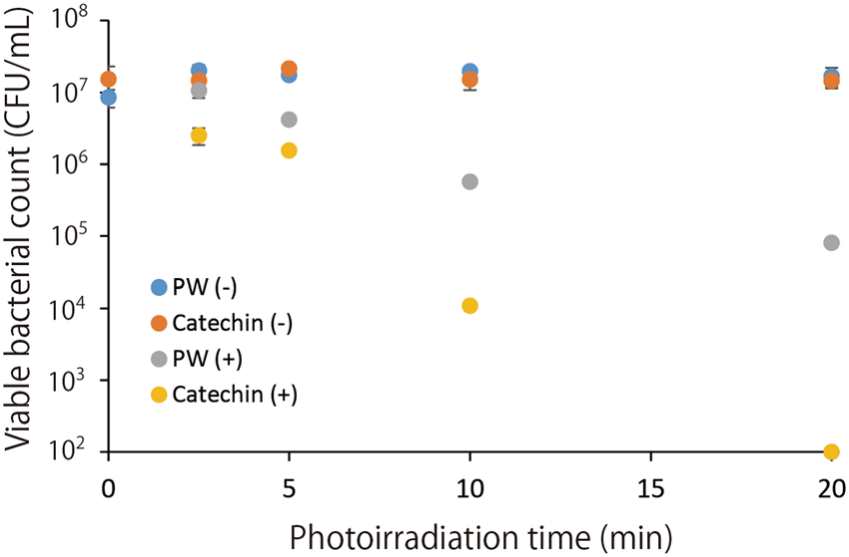
The bactericidal effect of the photoirradiation of (+)-catechin (1 mg/mL) on *Staphylococcus aureus*. Each value is the mean with the standard deviation (n = 3). PW (−): pure water without photoirradiation, Catechin (−): catechin without photoirradiation, PW (+): pure water with photoirradiation, Catechin (+): catechin with photoirradiation.

The result of the bactericidal assay against *Staphylococcus aureus* is summarized in Fig. 4. Without photoirradiation, the (+)-catechin solution kept in a light-shielding box for 2.5, 5, 10, and 20 min showed almost no bactericidal activity in comparison with that of the corresponding pure water groups. Photoirradiation alone showed a slight bactericidal activity. Namely, the photoirradiation of pure water for 10 and 20 min showed an approximate 1.5- and 2.3-log reduction of viable bacterial counts, respectively, compared with the corresponding pure water groups without photoirradiation. Furthermore, photoirradiation of the (+)-catechin solution effectively killed the bacteria in an irradiation time dependent manner, and 10 and 20 min of irradiation resulted in an approximate 3-log and a >5-log reduction, respectively.

In the experiment for examining the effect of thiourea, a ·OH scavenger, thiourea prominently attenuated the bactericidal activity of (+)-catechin upon photoirradiation (Fig. S1b).

### Liquid chromatography electrospray ionization mass spectrometric (LC-ESI-MS) and nuclear magnetic resonance (NMR) analyses for photoirradiated (+)-catechin

Representative LC chromatograms and summarized MS analyses are shown in Fig. 5. Concomitantly with decreases in the peak 2 that corresponded to (+)-catechin with irradiation time, increases in the two peaks 1 and 3 whose molecular formula was C_15_H_12_O_6_, as assessed using high resolution (HR)-ESI-MS, were observed. The [M + H]^+^ *m/z* of peak 2 was 291.0863 to 291.0876 (consistent with a calculated value of [M + H]^+^ *m/z* 291.0869 for C_15_H_15_O_6_), and those of peak 1 and 3 were 289.0716 to 289.0719 and 289.0727 to 289.0730 (consistent with a calculated value of [M + H]^+^ *m/z* 289.0712 for C_15_H_13_O_6_), respectively. The ^1^H and ^13^C NMR spectral data of the peak 1 and 3 are listed in Tables 1 and 2, respectively. The NMR spectra of the peak 1 and 3 are shown in Figs S3–S12 and Figs S13–S22, respectively.

**Figure 5:**
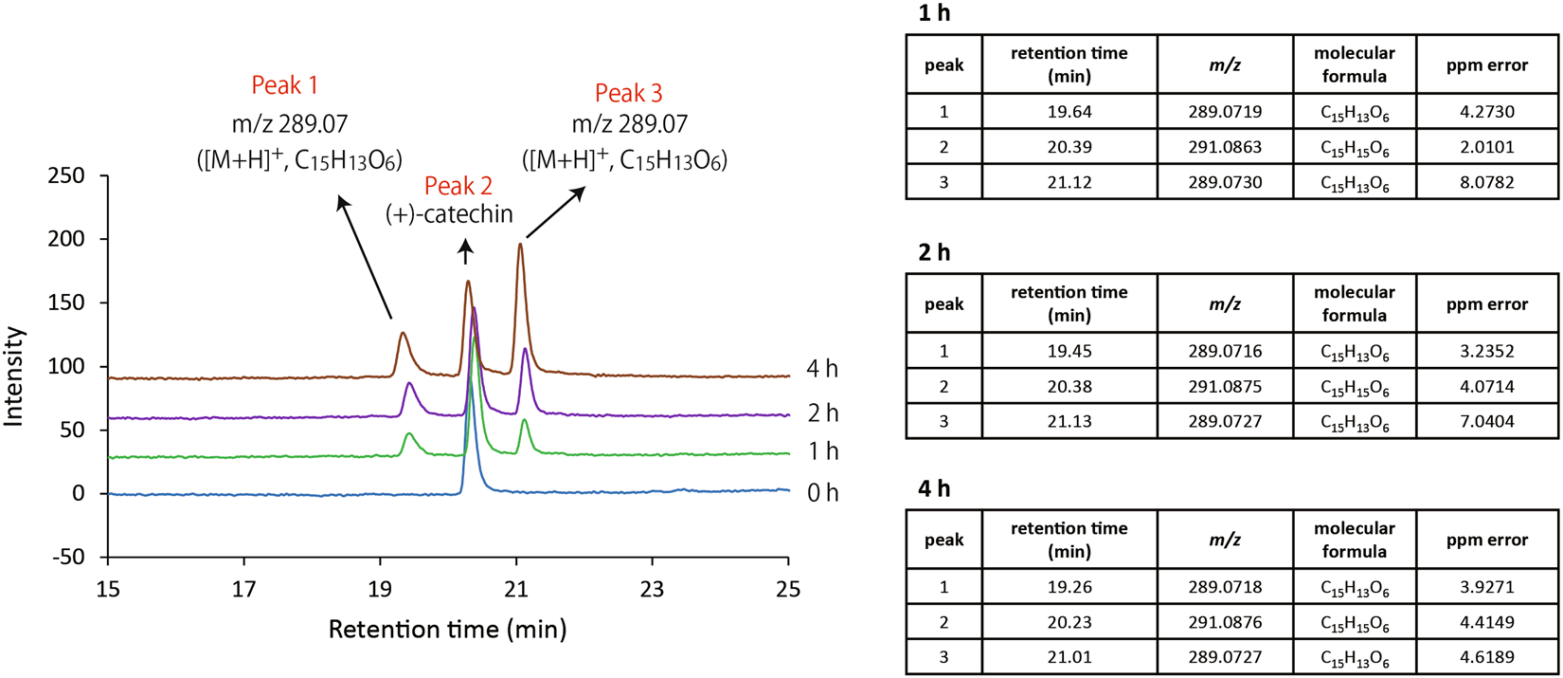
Representative LC chromatograms of photoirradiated (+)-catechin with *m/z* values, molecular formulas of [M + H]^+^, and the other detailed information analyzed using HRMS. Photoirradiation was carried out for 0–4 h. LC and HRMS stand for liquid chromatography and high-resolution mass spectrometry, respectively.

**Table 1:** NMR spectra of the peak 1 compound.

Position	δ_C_, mult	δ_H_, (int., mult., *J* in Hz)	^1^H-^1^HCOSY	HMBC (H→C)
**Peak 1 (in CD**_**3**_ **OD)**				
2	82.6, CH	5.39 (1H, d, 1.7)	H-3	C-3, C-4, C-9, C-1’, C-2’
3	66.7, CH	4.04 (1H, ddd, 9.0, 1.0, 1.0)	H-2, H-4a, H-4b	
4	37.5, CH_2_	a: 1.39 (1H, dd, 13.2, 2.5) b: 2.65 (1H, dd, 13.2, 9.3)	H-3, H-4b H-3, H-4a	C-3, C-6’ C-5, C-10
5	173.9, C			
6	−, CH	—		
7	−, C			
8	99.2, CH	5.48 (1H, s)		C-9, C-10
9	175.0, C			
10	48.0, C			
1’	127.5, C			
2’	112.2, CH	6.89 (1H, s)		C-2, C-4’, C-6’
3’	147.6, C			
4’	145.8, C			
5’	112.9, CH	6.81 (1H, s)		C-10, C-1’, C-3’, C-4’
6’	127.1, C			
**Peak 1 (in DMSO-** ***d*** **6)**				
2	80.3, CH	5.33 (1H, d, 1.5)	H-3	C-3, C-4, C-9, C-2’
3	65.7, CH	3.87 (1H, d, 9.0)	H-2, H-4a, H-4b	C-2
4	35.8, CH_2_	a: 1.22 (1H, d, 7.2) b: 2.42 (1H, dd, 9.6, 9.6)	H-3 H-3	C-3, C-6’
5	163.1, C			
6	−, CH	5.44, s		
7	−, C			
8	−, CH	5.24 (1H, s)		C-9
9	170.9, C			
10	48.6, C			
1’	126.2, C			
2’	112.2, CH	6.86 (1H, s)		C-2, C-1’, C-3’
3’	144.3, C			
4’	145.8, C			
5’	111.1, CH	6.67 (1H, s)		C-4’, C-6’
6’	125.6, C

**Table 2:** NMR spectra of the peak 3 compound.

Position	δC, mult	δH, (int., mult., *J* in Hz)	^1^H-^1^HCOSY	HMBC (H→C)
**Peak 3 (in CD**_**3**_ **OD)**				
2	79.7, C	5.32 (1H, d, 4.0)	H-3	C-3, C-4, C-9, C-2’
3	66.3, CH	4.38 (1H, ddd, 8.4, 4.0, 4.0)	H-2, H-4a, H-4b	C-1’
4	38.2, CH_2_	a: 1.86 (1H, dd, 13.0, 4.0) b: 2.10 (1H, dd, 13.0, 8.8)	H-3 H-3	C-3, C-9, C-10, C-6’ C-2, C-9, C-10
5	192.7, C			
6	82.6, CH	5.39, 5.48 (weak)		C-7, C-10
7	175.8, C			
8	98.5, CH	5.40 (1H, s)		C-6, C-9, C-10
9	173.8, C			
10	49.0, C			
1’	126.3, C			
2’	114.7, CH	6.92 (1H, s)		C-2, C-3’, C-4’, C-6’
3’	147.6, C			
4’	146.1, C			
5’	111.7, CH	6.85 (1H, s)		C-10, C-1’, C-4’
6’	127.5, C			
**Peak 3 (in DMSO-** ***d*** **6)**				
2	77.3, C	5.26 (1H, d, 4.2)	H-3	C-3, C-4, C-9, C-1’ C-2’
3	64.5, CH	4.22 (1H, ddd, 8.7, 4.2, 4.0)	H-2, H-4a, H-4b	
4	36.7, CH_2_	a: 1.62 (1H, dd, 12.7, 4.0) b: 1.97 (1H, dd, 12.7, 8.7)	H-3 H-3	C-5, C-10, C-6’ C-10, C-6’
5	175.9, C			
6	102.1, CH	5.41 (1H, s)		C-5, C-10
7	163.4, C			
8	98.4, CH	5.19 (1H, s)		C-6, C-9, C-10
9	169.5, C			
10	47.0, C			
1’	126.1, C			
2’	113.9, CH	6.82 (1H, s)		C-2, C-1’, C-3’
3’	145.8, C			
4’	144.3, C			
5’	110.5, CH	6.68 (1H, s)		C-10, C-4’, C-6’

**Figure 6:**
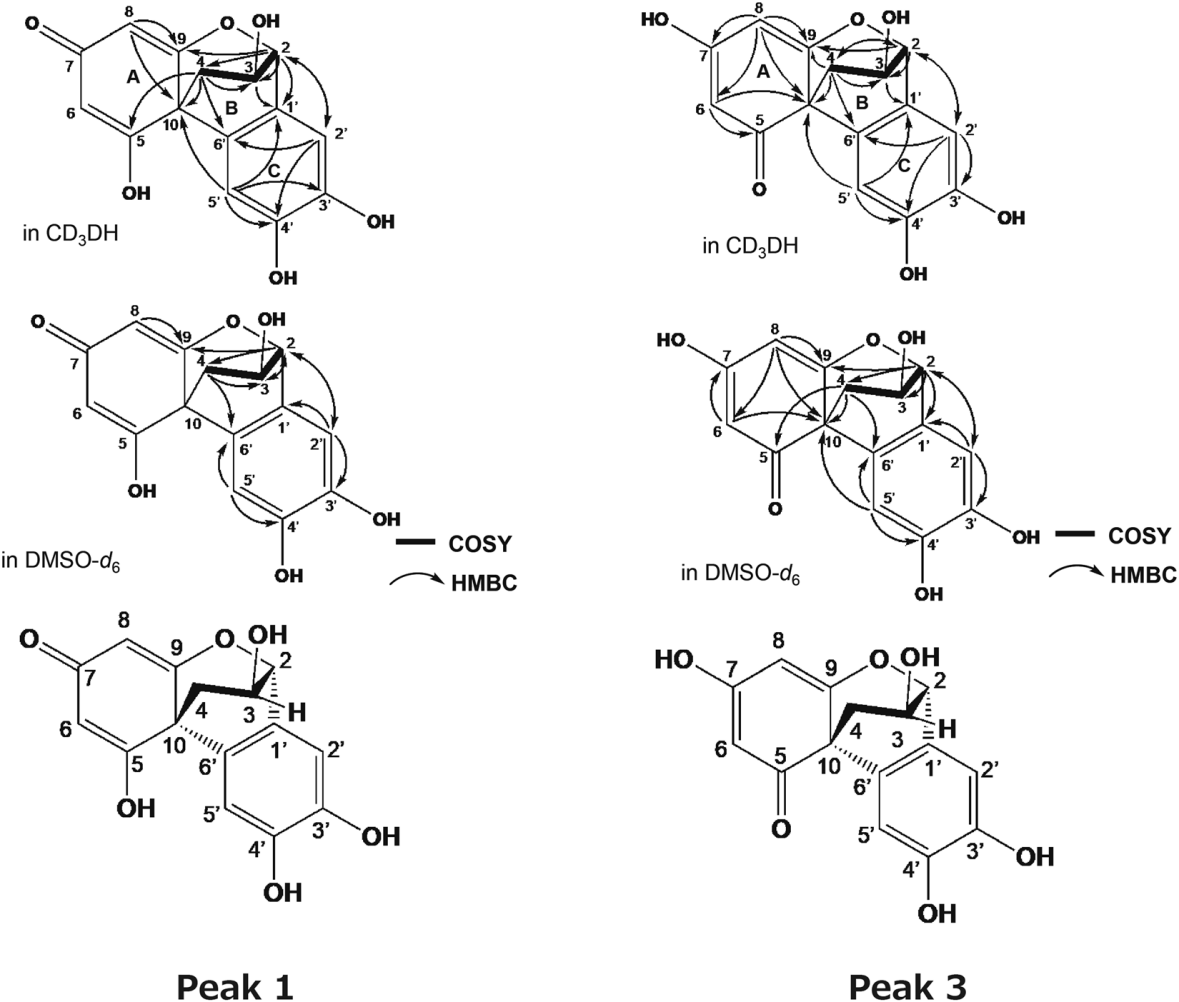
^1^H–^1^H COSY (bold) and selected HMBC (arrow) correlations of the peak 1 and 3. COSY and HMBC stand for correlation spectroscopy and heteronuclear multiple bond coherence, respectively.

In the peak 3, the ^1^H NMR data indicate the presence of an oxygenated *sp*^3^ methine proton, four *sp*^2^ methine protons and a methylene group. The ^13^C NMR spectrum shows the resonances of 15 carbons, which were classified into 10 olefinic carbons containing four oxygenated quaternary carbons, two oxygenated *sp*^3^ methine carbons, a *sp*^3^ quaternary carbon, a *sp*^3^ methylene carbon, and a carbonyl carbons at δc 192.7 in CD_3_OH by heteronuclear multi quantum correlation (HMQC) spectra. The ^1^H–^1^H correlation spectroscopY (COSY) indicated the presence of one partial structure, C-2/C-4, as shown in Fig. 6. Each ring unit was identified based on the following heteronuclear multiple bond coherence (HMBC) correlations: A-ring in CD_3_OD, from H-6 to C-5 and C-10; H-8 to C-6, C-7, C-9, and C-10: B-ring, from H-2 to C-3, C-4 and C-9; from H-3 to C-1′; from H-4 to C-2, C-3, C-9, C-10, and C-6′: C-ring, from H-2′ to C-3′, C-4′ and C-6′; from H-5′ to C-1′, C-4′ and C-6′. The connections of A, B and C-ring were confirmed by HMBC correlations from H-6 to C-10; from H-8 to C-9 and C-10; from H-2 to C-9 and C-2′; H-2′ to C-2 and C-6′; from H-5′ to C-10 and C-1′ (Fig. 6).

**Figure 7:**
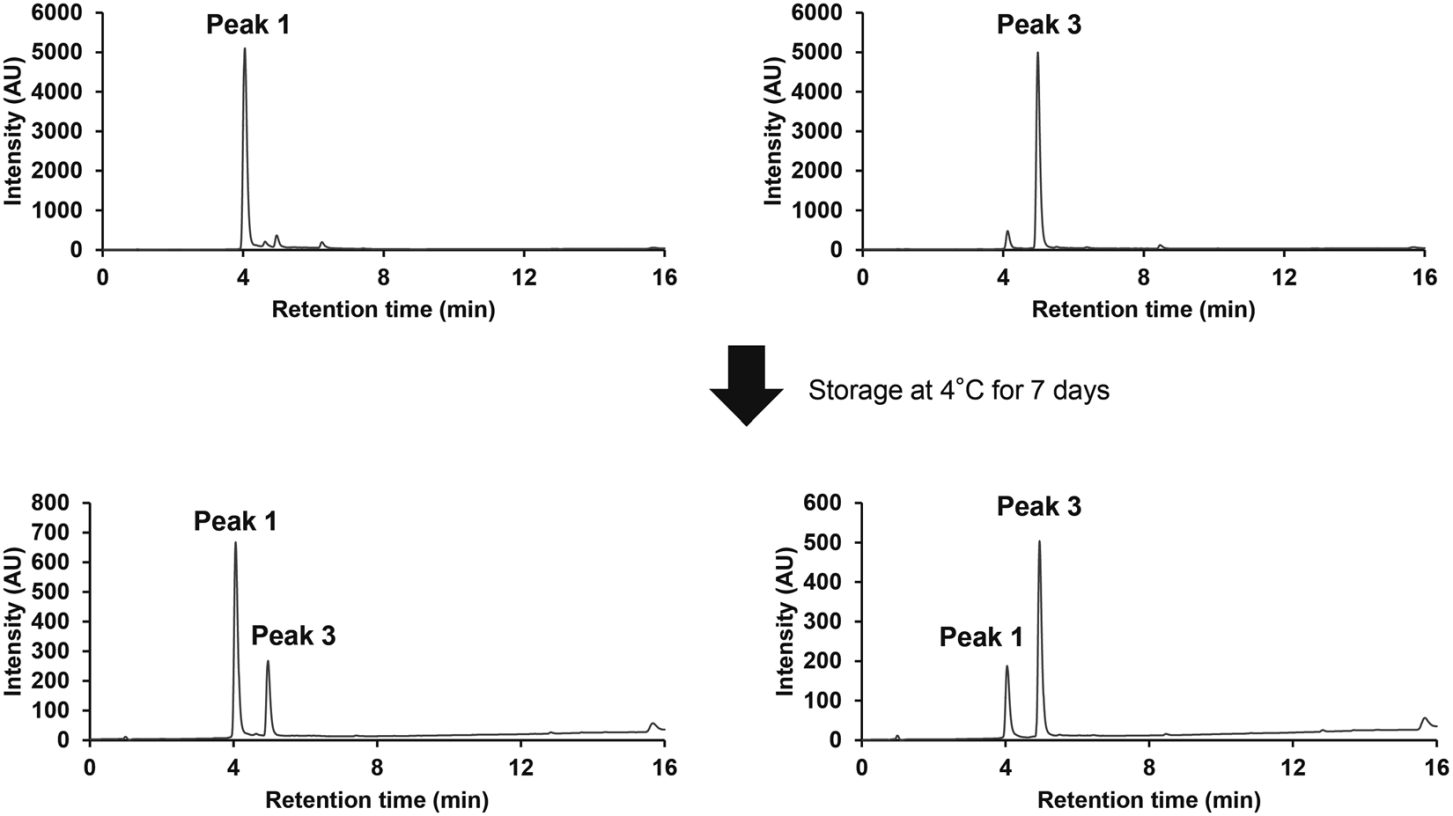
HPLC analyses of tautomerization between the peak 1 and 3. The peak 1 and 3 were isolated by chromatographic procedures and was stocked at 4 °C for 7 days. HPLC stands for high performance liquid chromatography.

**Figure 8:**
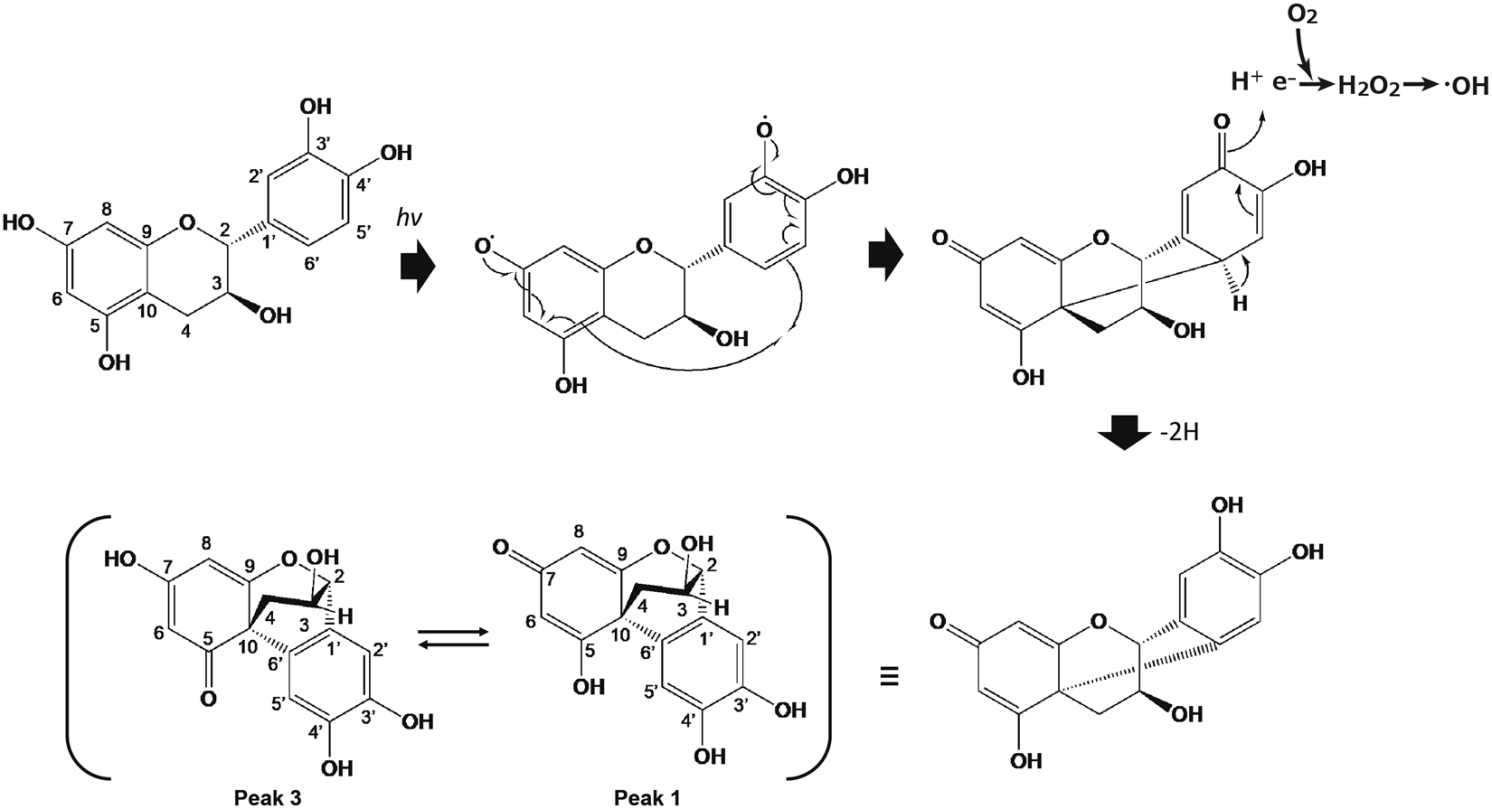
Proposed pathway of intramolecularly cyclized derivative formation from photoirradiated (+)-catechin coupled with ROS generation. ROS stands for reactive oxygen species.

In the peak 1, the ^1^H NMR data indicated the presence of two oxygenated *sp*^3^ methine protons, three *sp*^2^ methine protons and a methylene. The ^13^C NMR spectrum showed the resonances of 13 carbons, which were classified into nine olefinic carbons containing four oxygenated quaternary carbons, two oxygenated *sp*^3^ methine carbon, a *sp*^3^ quaternary carbon, a *sp*^3^ methylene carbon by HMQC spectra. The ^1^H–^1^H COSY indicated the presence of one partial structure, C-2/C-4 as shown in Fig. 6. B,C ring units were identified based on the following HMBC correlations: B-ring, from H-2 to C-3, C-4, C-9 and C-1′; from H-4 to C-3, C-5, C-10, and C-6′: C-ring, from H-2′ to C-4′ and C-6′; from H-5′ to C-1′, C-3′, C-4′ and C-6′. The connections of B and C-ring were confirmed by HMBC correlations from H-2 to C-2′; H-2′ to C-2 and C-6′; from H-5′ to C-10 C-1′ and C-6′ (Fig. 6). However, the structure elucidation of peak 1 was difficult due to poor NMR signals, especially ring A, as shown in Table 1, Figs S8 and S12. It was considered that the absence of signals at 6 and 7 positions was attributable to a tautomerization. The peak 1 and 3 were isolated by chromatographic procedures and was stored at 4 °C for 7 days. The LC analysis of one storage sample revealed the presence of the peak in the other storage sample (Fig. 7) and two compounds equilibrated over time (Figs 8, S23 and S24). As shown in Fig. 6, the planter structure of peak 1 was elucidated by comparisons with NMR spectra of peak 3 and 2,4′,5′,12-tetrahydroxy-9,10-benzo-7-oxatricyclo[6.2.2.01,6]dodeca-2,5,9-trien-4-one (termed as the irradiation product). The irradiation product is a compound generated by a radical reaction of (+)-catechin and 2,2′-azobis(2-methylpropionitrile) via irradiation with fluorescent lamps at 40 °C for 10 days as reported in a previous study^19^. The physico-chemical properties of this irradiation product were remarkably similar to that of peak 1 (Table S1). Finally, the absolute configuration of the irradiation product was determined by X-ray crystallography^19^. The absolute configuration of peak 1 is most likely the same as the irradiation product from good agreement with optical rotation of both compounds. In addition, these two cyclized compounds have methine and methylene, but not methyl group. Therefore, the carbon with proton of the peak 1 could be sufficiently assigned by a heteronuclear multiple quantum correlation (HMQC) spectrum (Fig. S10). Accordingly, it is strongly suggested that the generated cyclization products be a keto-enol tautomer.

## Discussion

The present study demonstrated that photoirradiation of (+)-catechin has significant prooxidative potential, and can exert a bactericidal effect against *S. aureus* with a >5-log reduction in viable counts within 20 min. ROS analyses showed that ·OH and H_2_O_2_ were generated through the photoirradiation of (+)-catechin, suggesting that the potent oxidative power of ROS, especially ·OH, is pivotal for bactericidal action, as reported previously^14–16,20–22^. This was also supported by the experiment in which the effect of thiourea, a ·OH scavenger, was examined (Fig. S1b). That is, the bactericidal effect was prominently attenuated by thiourea, suggesting that the major contributor to the bactericidal action of (+)-catechin upon photoirradiation be ·OH. Involvement of H_2_O_2_ itself in the bactericidal action would be neglected because it was confirmed that exposure to 50 or 100 μM H_2_O_2_ for 20 min did not show any bactericidal effect (data not shown). Although there would be a possibility for the two cyclized products (Peak 1 and 3) to exert bactericidal action, the effect would also be negligible in the present study because the bactericidal effect of (+)-catechin upon photoirradiation was almost completely canceled by the ·OH scavenger. The ·OH yield, expressed as DMPO-OH, increased with photoirradiation time up to approximately 150 s and thereafter reached a plateau, whereas H_2_O_2_ yield increased with time at least up to 20 min. Since the prior photoirradiation of (+)-catechin without DMPO for 600 s did not affect the DMPO-OH yield produced by the subsequent irradiation for 150 s with DMPO, it is suggested that ·OH was continuously generated, at least during photoirradiation for 600 s. Provided that DMPO-OH was linearly generated within the first 19 s of photoirradiation, the total yield of·OH in 10 min would be approximately 20 μM. Accordingly, it was postulated that DMPO-OH was degraded or converted to an ESR silent compound by the newly formed· OH or a reductive reaction coupled with the photooxidation of (+)-catechin. To verify this hypothesis, we examined if DMPO-OH would be decayed during the photoirradiation of (+)-catechin. Levels of DMPO-OH pre-formed through water sonolysis decreased gradually with photoirradiation time, and after 150 s the concentration reached an almost undetectable level. Since our previous study also revealed that DMPO-OH formed by photoirradiation of (+)-catechin was stable at least for approximately 10 min^22^, DMPO-OH was likely degraded to become an ESR silent compound during the photoirradiation of (+)-catechin. As such, the proposed reaction (i) is expressed as *v* = −d[DMPO-OH]/dt = *k*_1_[DMPO-OH] in which *v* and *k*_1_ indicate the velocity of DMPO decay and the first order rate constant, respectively.

1 $$[\mathrm{DMPO} \mbox{-} \mathrm{OH}]\to [{\rm{ESR}}\,{\rm{silent}}\,{\rm{compound}}]$$

2 $$-{\rm{d}}[\mathrm{DMPO} \mbox{-} \mathrm{OH}]/{\rm{dt}}={k}_{1}[\mathrm{DMPO} \mbox{-} \mathrm{OH}]$$

When the equation (3), which derives from equation (2) is integrated, the equation (4), in which [DMPO-OH]_0_ indicates the initial concentration of [DMPO-OH], is obtained.

3 $$-{\rm{d}}[\mathrm{DMO} \mbox{-} \mathrm{OH}]/[\mathrm{DMPO} \mbox{-} \mathrm{OH}]={k}_{1}{\rm{dt}}$$

4 $$\mathrm{ln}([\mathrm{DMPO} \mbox{-} \mathrm{OH}]/{[\mathrm{DMPO} \mbox{-} \mathrm{OH}]}_{0})=-\,{k}_{1}t$$

As shown in Fig. 3c, the semi-log plot is linear with a slope value of 0.0203 indicating that the first order rate constant *k*_1_ = 0.0203/s, and accordingly *t*_1/2_, calculated from the equation (4) in which [DMPO-OH] becomes [DMPO-OH]_0_/2 with *t* = *t*_1/2_, is 34.1 s. Therefore, it is suggested that DMPO-OH is exponentially decayed by photoirradiation of (+)-catechin according to an exponential function, [DMPO-OH] = [DMPO-OH]_0_ · *e*^−*k*1*t*^.

As for autoxidation of catechins, it has been reported that autoxidation rate of catechins is pH dependent^10^. According to the study, this pH dependency is not simply interpreted from acid dissociation constant (p*K*a) of the phenolic group, but the stability of O_2_^−^·, which is generated by one electron reduction of molecular oxygen coupled with oxidation of the phenolic group and proceeds further autoxidation of catechins, is involved. That is, since the stability of O_2_^−^· increases with an increase in pH^23^, the increased stability of O_2_^−^· also seems to be responsible for the pH dependence of autoxidation. In the present study, pH of catechin aqueous solution at 1 mg/mL was 5.4, so that only trace level of H_2_O_2_ was generated within 20 min, indicating that autoxidation was not accelerated under such a low pH condition. By contrast, photoirradiation of the pH unadjusted (+)-catechin solution yielded around 50 μM H_2_O_2_ for 20 min, indicating that photon energy would have an ability to accelerate oxidation of the phenolic group of (+)-catechin even under acidic conditions. This would be one of the major differences between autoxidation and photooxidation of catechins. Regarding the photooxidation process of polyphenols, no clear evidence for the structural and conformational changes of the polyphenols has been reported to date. In the LC/MS analysis of the photoirradiation of (+)-catechin in the present study, the peak of (+)-catechin with *m/z* 291.07 ([M + H]^+^ with molecular formula C_15_H_15_O_6_) decreased with time, whereas the two peaks with *m/z* 289.07 ([M + H]^+^ with molecular formula C_15_H_13_O_6_) increased. Subsequent NMR analysis revealed that the two peaks corresponded to intramolecularly cyclized derivatives that were converted from an oxidized form of (+)-catechin. In the case of autoxidation of catechin, it has been postulated that the B ring of (+)-catechin is oxidized to quinone with H_2_O_2_ generation via a semiquinone intermediate with O_2_^−^· generation. It is also well known that the enzymatic browning of apples is caused by polyphenol oxidase (PPO) with polyphenols as its substrates. As shown in Fig. S25, contact of polyphenols with PPO results in oxidation of the polyphenols to the corresponding quinones through the action of PPO in the presence of oxygen, and subsequently the quinones formed are automatically polymerized to form brown polymers^24^. As such, the end products of not only the autoxidation process of catechins, as proposed previously^10,11^, but also the PPO-mediated oxidation of catechins, are quinones. Our present study clearly showed that the photooxidation process of (+)-catechin is different from the autoxidation and enzymatic oxidation processes. That is, two intramolecularly cyclized derivatives were identified as the end products of the photooxidation of (+)-catechin. We conducted a literature search and found that only one paper described a novel intramolecular cyclization product of (+)-catechin, 2,4′,5′,12-tetrahydroxy-9,10-benzo-7-oxatricyclo[6.2.2.0^1,6^]dodeca-2,5,9-trien-4-one^19^. In that study, the compound was obtained from a radical scavenging reaction of (+)-catechin with 2,2′-azobis(2-methylpropionitrile), and NMR analysis showed that the B and C rings of (+)-catechin were maintained with the presence of a substituent on the 6′-position carbon in the B ring. Based on the NMR and the other general analyses of the present study, we propose a pathway to form intramolecularly cyclized derivatives from the photoirradiation of (+)-catechin (Fig. 8) and the presence of a keto-enol tautomer. Regarding the cyclization favorably occurs through the B ring, as speculated from the previous studies^7,9–11^, the electron-donating property of the B ring, especially dihydroxyl groups (catechol moiety), would be more potent than the other two rings. Since the C-C bond formation is irreversible, even if the B ring oxidation product could be produced during the photoirradiation, the product might be attacked by generated ·OH and finally formed as more stable C-C bond product.

## Methods

### Reagents

Reagents were purchased from the following sources: (+)-catechin from Tokyo Chemical Industry (Tokyo, Japan); DMPO from Labotec (Tokyo, Japan); catalase and H_2_O_2_ from Wako Pure Chemical Industries (Osaka, Japan); and 4-hydroxy-2,2,6,6-tetramethylpiperidine *N*-oxyl (TEMPOL) from Sigma-Aldrich (St. Louis, MO, USA). All other reagents used were of analytical grade.

### Light source

An experimental device equipped with a light emitting diode with a wavelength of 400 nm (NHH105UV, Lustrous Technology, Shiji, Taiwan) was used. The output power of the LED, measured using a power meter (FieldMate, Coherent, Santa Clara, CA, USA), was set at 400 mW per LED corresponding to an irradiance of 130 mW/cm^2^ at a distance of 15 mm from the LED. A four-sided, clear methacrylate plastic cuvette containing the sample was placed in the experimental device. LED-light irradiation was performed on both sides of the plastic cuvette (total irradiance: 260 mW/cm^2^).

### Colorimetric determination of H_2_O_2_ and ESR analysis of ·OH

(+)-Catechin was dissolved to be 1 mg/mL in pure water without pH adjustment, and pH of the solution was around 5.4. For H_2_O_2_ determination, 500 μL of (+)-catechin solution (1 mg/mL) in a plastic cuvette was photoirradiated for 2.5, 5, 10, and 20 min. Immediately after irradiation, the H_2_O_2_ concentration was determined using a colorimetric method based on the peroxide-mediated oxidation of Fe^2+^ followed by the reaction of Fe^3+^ with xylenol orange^25^. To confirm if the generated H_2_O_2_ responds to the catalytic action of catalase, 500 μL of (+)-catechin solution (1 mg/mL) photoirradiated for 20 min was mixed with 500 μL of 0.1 M Na-K phosphate buffer (PB, pH 7.4) or catalase solution (5000 U/mL in 0.1 M PB), and the H_2_O_2_ concentration was similarly determined.

Qualitative and quantitative analyses of ·OH generated during the photoirradiation of (+)-catechin were performed using an ESR spin trapping technique, as reported previously^22^. An aliquot (483 μL) of (+)-catechin solution was mixed with 17 μL of DMPO in a plastic cuvette to reach a final concentration of 1 mg/mL and 300 mM for (+)-catechin and DMPO, respectively. Then, the sample was photoirradiated for 0, 9, 19, 38, 75, 150, 300, and 600 s. After irradiation, the sample was transferred to a quartz cell for ESR spectrometry, and the ESR spectrum was recorded using an X-band ESR spectrometer (JES-FA-100, JEOL, Tokyo, Japan). The measurement conditions for ESR were as follows: field sweep, 331.89–341.89 mT; field modulation frequency, 100 kHz; field modulation width, 0.1 mT; amplitude, 200; sweep time, 2 min; time constant, 0.03 s; microwave frequency, 9.420 GHz; and microwave power, 4 mW. TEMPOL (2 µM) was used as a standard to calculate the concentration of spin-trapped radicals, and the ESR spectrum of manganese held in the ESR cavity was used as an internal standard.

Because the DMPO-OH yield reached a plateau after 150 s of photoirradiation, the following experiment was conducted to examine whether ·OH was continuously generated during photoirradiation for 600 s. An aliquot (483 μL) of (+)-catechin solution was photoirradiated for 600 s, and then 17 μL of 8.9 M DMPO was added to the photoirradiated sample to reach a final concentration of 1 mg/mL and 300 mM for (+)-catechin and DMPO, respectively. Immediately after the addition of DMPO, the sample was further photoirradiated for 150 s. Then, ESR analysis was performed as described above.

### Effect of photoirradiation of (+)-catechin on DMPO-OH generated by water sonolysis

The experimental device for ultrasound generation was identical to that used in our previous study^26^, and the experimental scheme is illustrated in Fig. S26. The ultrasound power was 30 W and the frequency was 1.65 MHz. A glass tube (15 mm in diameter and 85 mm long) containing a sample was set into the device so that the sample was exposed to the ultrasound irradiation from the transducer at the bottom. The temperature of the water bulk was controlled at 24 ± 1 °C. An aliquot (500 μL) of pure water with 20 mM DMPO was subjected to ultrasound irradiation for 15 s. Then, 250 μL of the irradiated solution was mixed with the same volume of 2 mg/mL of (+)-catechin in a plastic cuvette, followed by photoirradiation for 0 to 150 s. Immediately after photoirradiation, the ESR analysis for DMPO-OH was performed as described above.

### Bactericidal assay

*S. aureus* JCM 2413 purchased from the Japan Collection of Microorganisms, RIKEN BioResource Center (Wako, Japan) was used. A suspension of *S. aureus* was prepared in sterile physiological saline from a culture grown on brain heart infusion (BHI) agar (Becton Dickinson Labware, Franklin Lakes, NJ, USA) aerobically at 37 °C overnight. In a plastic cuvette, 450 µL of (+)-catechin solution or pure water was mixed with 50 µL of the bacterial suspension to reach a final concentration of 1 mg/mL and approximately 10^7^ colony forming units (CFU)/mL for (+)-catechin and *S. aureus*, respectively. Then, the samples were photoirradiated for 2.5, 5, 10, and 20 min. After irradiation, 50 µL of the sample was mixed with an equal volume of sterile catalase solution (5000 U/mL phosphate buffer [pH 7.4]) to terminate the bactericidal effect of the H_2_O_2_ generated by the photoirradiated (+)-catechin. A 10-fold serial dilution of the mixture was prepared using sterile physiological saline, and 10 µL of the diluted solution was seeded onto a BHI agar plate. The agar plates were cultured as described above for 2 d, and the CFU/mL was determined. In addition, as controls, samples were maintained for 2.5, 5, 10, and 20 min in a light-shielding box, instead of being photoirradiated, and subjected to the same procedures.

To examine if the bactericidal action of (+)-catechin upon photoirradiation would be attributable to ·OH, thioure that is an established means of mitigating the effects of ·OH damage in both eukaryotes and prokaryotes as a potent ·OH scavenger^27–30^ was added to the reaction mixture. The reaction mixture consisting of 425 μL of (+)-catechin solution, 50 μL of the *S. aureus* suspension and 25 μL of thiourea was prepared to reach final concentrations of 1 mg/mL for (+)-catechin, approximately 10^7^ CFU/mL for the bacteria, and 150 mM for thiourea. Then, the sample was photoirradiated for 10 min. The CFU was determined after each treatment as described above.

### LC-ESI-MS, NMR, and the other general experimental analyses for photoirradiated (+)-catechin

An aliquot (500 μL) of 1 mg/mL (+)-catechin solution in a plastic cuvette was photoirradiated for 0, 1, 2, and 4 h. Following passage through a filter (polyvinylidene difluoride; pore size, 0.2 μm), the resultant sample was injected into the electrospray ion source of the QSTAR ESI quadruple time-of-flight mass spectrometer (AB Sciex; Framingham, MA, USA) coupled to an Agilent 1200 series (Agilent Technologies, Santa Clara, CA, USA). Chromatographic separation was undertaken using an Inertsil ODS-4 (3.0 × 250 mm, GL Sciences, Tokyo, Japan) at 40 °C. With regard to gradient elution, solvent A was water with 0.1% formic acid, and solvent B was methanol with 0.1% formic acid. The gradient elution was 0–30 min and 5–100% B. The flow rate was 0.5 mL/min, the injection volume was 5 μL, and detection occurred at 254 nm using a photodiode array detector. ESI/MS was recorded for 30 min in the *m/z* region from 100 to 2000 Da with the following instrument parameters: ion spray voltage = 5500 V, source gas = 50 L/min, curtain gas = 30 L/min, declustering potential = 50 V, focusing potential = 250 V, temperature = 450 °C, and detector voltage = 2300 V. LC/MS analyses were undertaken using high-resolution ESI-MS (R ≥ 10,000; tolerance for mass accuracy = 5 ppm).

For NMR analysis, fractions were collected from the chromatographic separation of a photoirradiated (+)-catechin solution performed as described above except that an ODS column (Intertsil ODS-4, 14 × 250 mm, GL Sciences Inc.) was used at a flow rate of 7 mL/min. The collected fractions from the two peaks with *m/z* 289.07 ([M + H]^+^ with molecular formula C_15_H_13_O_6_) were concentrated to dryness *in vacuo*. NMR spectra were measured using a Varian XL-400 (Varian, Palo Alto, CA, USA), with ^1^H-NMR at 400 MHz and ^13^C-NMR at 100 MHz in CD_3_OD or DMSO-*d*_6_. The chemical shifts were expressed in ppm and referenced to residual CD_3_OD (3.31 ppm) or DMSO-*d*_6_ (2.48 ppm) in the ^1^H-NMR spectra and CD_3_OD (49 ppm) or DMSO-*d*_6_ (39.5 ppm) in the ^13^C-NMR spectra.

Infrared spectroscopy spectra (KBr) were taken on a Horiba FT-710 Fourier transform Infrared spectrometer. Ultraviolet spectra were measured with a Hitach U-2810 spectrophotometer. Optical rotation was measured on a JASCO model DIP-1000 polarimeter.

All other information and detailed data of NMR spectra can be found in Supplementary information.

## Electronic supplementary material

Supplementary information

## Data Availability

The datasets generated during and/or analysed during the current study are available from the corresponding author on reasonable request.
