# Supplementary material for: A novel pathway for the photooxidation of catechin in relation to its prooxidative activity
Source: Sci Rep. 2018 Aug 27;8:12888. doi: 10.1038/s41598-018-31195-x (PMC6110801; doi:10.1038/s41598-018-31195-x)
Supplement: Supplementary file 1 — Supplementary information [file 41598_2018_31195_MOESM1_ESM.pdf]

Supplementary Information

A novel pathway for the photooxidation of catechin in relation to, its prooxidative activity

Shunichi Shishido[1], Rei Miyano[2], Takuji Nakashima[2], Hirotaka Matsuo[2], Masato Iwatsuki[2], Keisuke Nakamura[1], Taro Kanno[1], Hiroshi Egusa[1], and Yoshimi Niwano[1,3]

[1]Tohoku University Graduate School of Dentistry, 4-1 Seiryo, Aoba-ku, Sendai 980-8575, Japan. [2]Kitasato Institute for Life Sciences, Kitasato University, 5-9-1 Shirokane, Minato-ku, Tokyo 108-8641, Japan. [3]Faculty of Nursing, Shumei University, 1-1 Daigaku-cho, Yachiyo, Chiba 276-0003, Japan.

Table S1 Physico-chemical properties and ^{1}H NMR data of peak 1 and 2,4′,5′,12-tetrahydroxy-9,10-benzo-7-oxatricyclo[6.2.2.01,6]dodeca-2,5,9-trien-4-one

		Peak 1	2,4′,5′,12-tetrahydroxy-9,10-benzo-7-oxatricyclo[6.2.2.01,6]dodeca-2,5,9-trien-4-one*
Molecular formula		$C_{15}H_{12}O_6$	$C_{15}H_{12}O_6$
Molecular weight		288	288
ESI-MS (*m/z*)	calcd. for $C_{15}H_{13}O_6$	289.0707	-
	found	289.0710	-
Elemental analysis	calcd. for $C_{15}H_{12}O_6 \cdot H_2O$	-	C, 58.83; H, 4.61% for
	found	-	C, 58.83; H, 4.87%
$[\alpha]_D^{21}$ (MeOH)		+ 94.5 (*c* 0.1)	$[\alpha]_D^{25}$ (MeOH) + 218.0 (*c* 0.0337)
UV λ_{max}^{MeOH} nm (ε)		206 (29,223), 248 (14,132), 285 (6,218)	211 (22,600), 248 (21,300), 286 (7,280)
IR ν (KBr) cm^{-1}		3325, 1655, 1616, 1516	3510, 3200, 2710, 1705, 1673

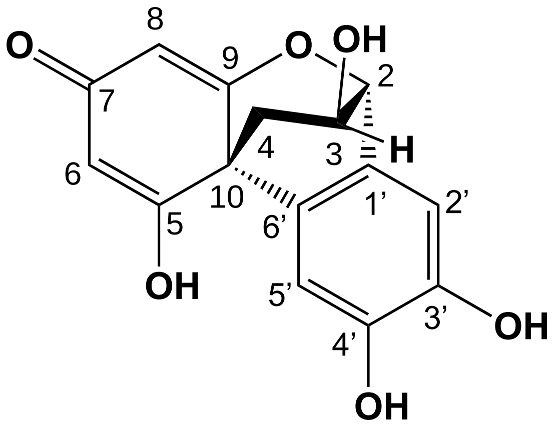

2,4′,5′,12-tetrahydroxy-9,10-benzo-7-oxatricyclo[6.2.2.01,6]dodeca-2,5,9-trien-4-one*

Reference compound (in CD_3DH)		
Position	δ_C, mul t	δ_H (int., mult., J in Hz)
2	82.6, CH	5.39 (1H, d, 1.7)
3	66.7, CH	4.04 (1H, ddd, 9.0, 1, 1)
4	37.5, CH_2	a : 1.39 (1H, dd, 13.2, 2.5)
		b : 2.65 (1H, dd, 13.2, 9.3)
5	173.9, C	
6	-, CH	-
7	-, C	
8	99.2, CH	5.48 (1H, s)
9	175.0, C	
10	48.0, C	
1'	127.5, C	
2'	112.2, CH	6.89 (1H, s)
3'	147.6, C	
4'	145.8, C	
5'	112.9, CH	6.81 (1H, s)
6'	127.1, C	

Reference compound (in CD_3DH)		
Position	δ_C, mul t	δ_H (int., mult., J in Hz)
2	82.6, CH	5.31 (1H, d, 1.5)
3	67.7, CH	3.95 (1H, ddd, 9.3, 2.6, 1.5)
4	37.4, CH_2	a : 1.29 (1H, dd, 13.4, 2.6)
		b : 2.55 (1H, dd, 13.4, 9.3)
5	176.6, C	
6	-, CH	-
7	190.6, C	
8	99.2, CH	5.40 (1H, s)
9	175.1	
10	49.6, C	
1'	127.0, C	
2'	112.9, CH	6.80 (1H, s)
3'	147.6, C	
4'	146.0, C	
5'	112.2, CH	6.71 (1H, s)
6'	127.4, C	

*Hirose Y., et al., A novel intermolecular cyclization product of (+)-catechin under radical reaction. Chemistry Letters, 21, 2361-2362 (1992), https://doi.org/10.1246/cl.1992.2361

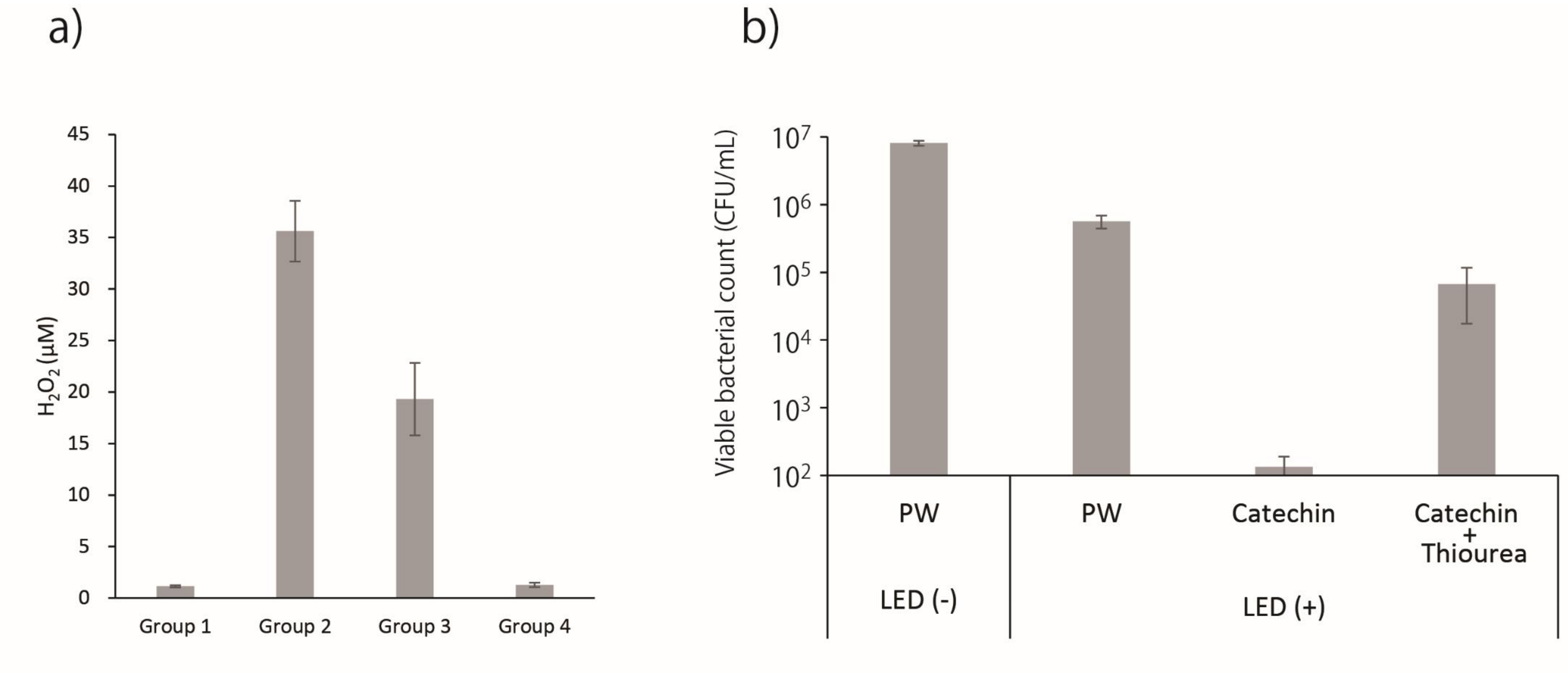

Fig. S1 a) Effect of catalase on H_2O_2 generated by photoirradiation of (+)-catechin. Group 1: 500 µL of (+)-catechin (1 mg/mL) kept under light shielding condition for 20 min, Group 2: 500 µL of (+)-catechin (1 mg/mL) photoirradiated for 20 min, Group 3: 500 µL of (+)-catechin (1 mg/mL) photoirradiated for 20 min + 500 µL of phosphate buffer (PB, pH 7.4), and Group 4: 500 µL of (+)-catechin (1 mg/mL) photoirradiated for 20 min + 500 µL of catalase (5000 U/mL in PB). Mean ± SD (n=4).
b) Influence of thiourea, a ･OH scavenger, on the bactericidal effect of the photoirradiation of (+)-catechin on *Staphylococcus aureus*. Mean ± SD (n=3).

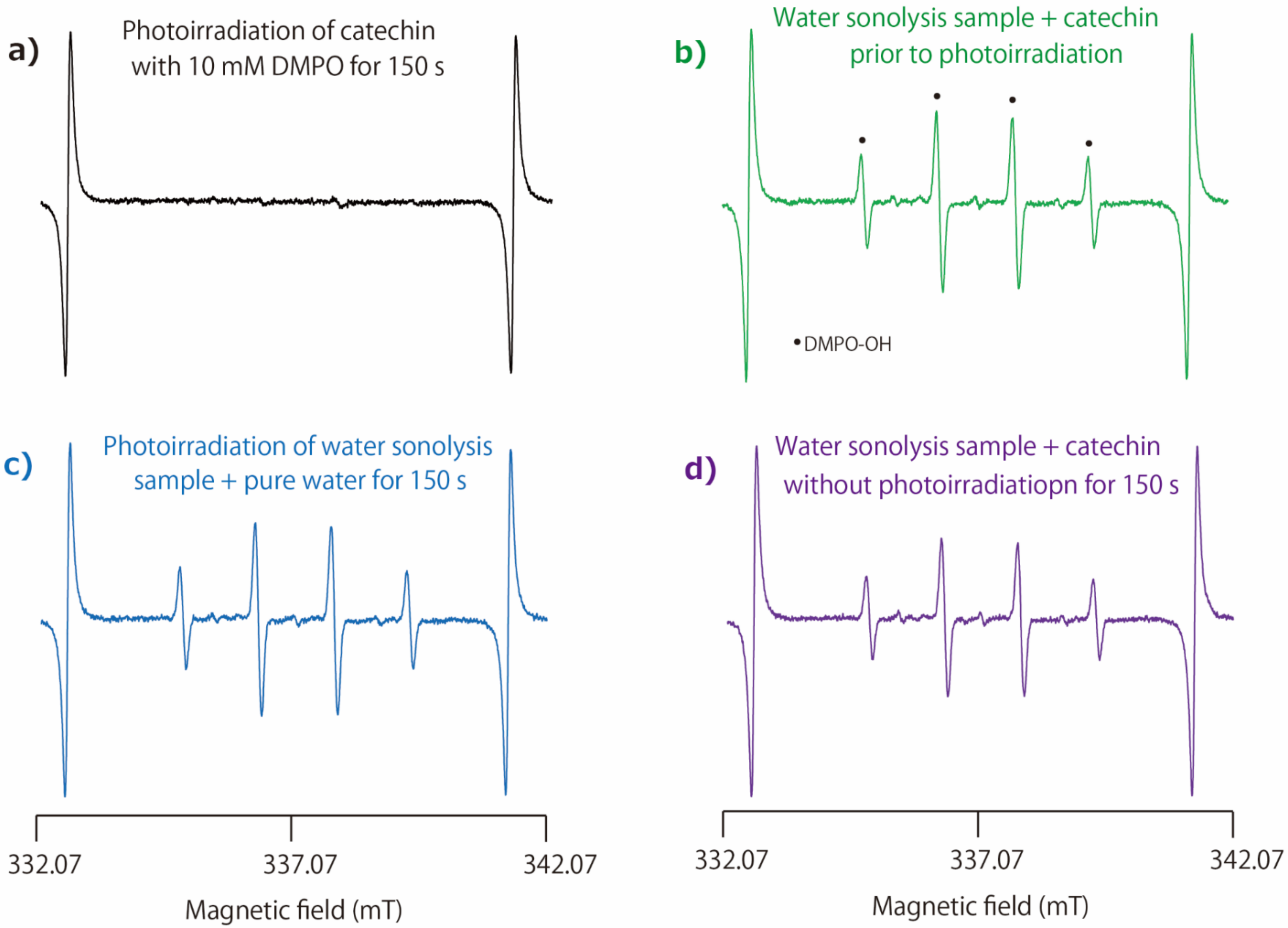

Fig. S2 Representative ESR spectra obtained from a) 500 µl of (+)-catechin (1 mg/mL) with 10 mM DMPO subjected to photoirradiation for 150s, b) 250 µL of water sonolysis sample + 250 µL of (+)-catechin (2 mg/mL) prior to photoirradiation, c) 250 µL of water sonolysis sample + 250 µL of pure water subjected to photoirradiation for 150s, and d) 250 µL of water sonolysis sample + 250 µL of (+)-catechin (2 mg/mL) kept under light-shielding condition for 150 s.

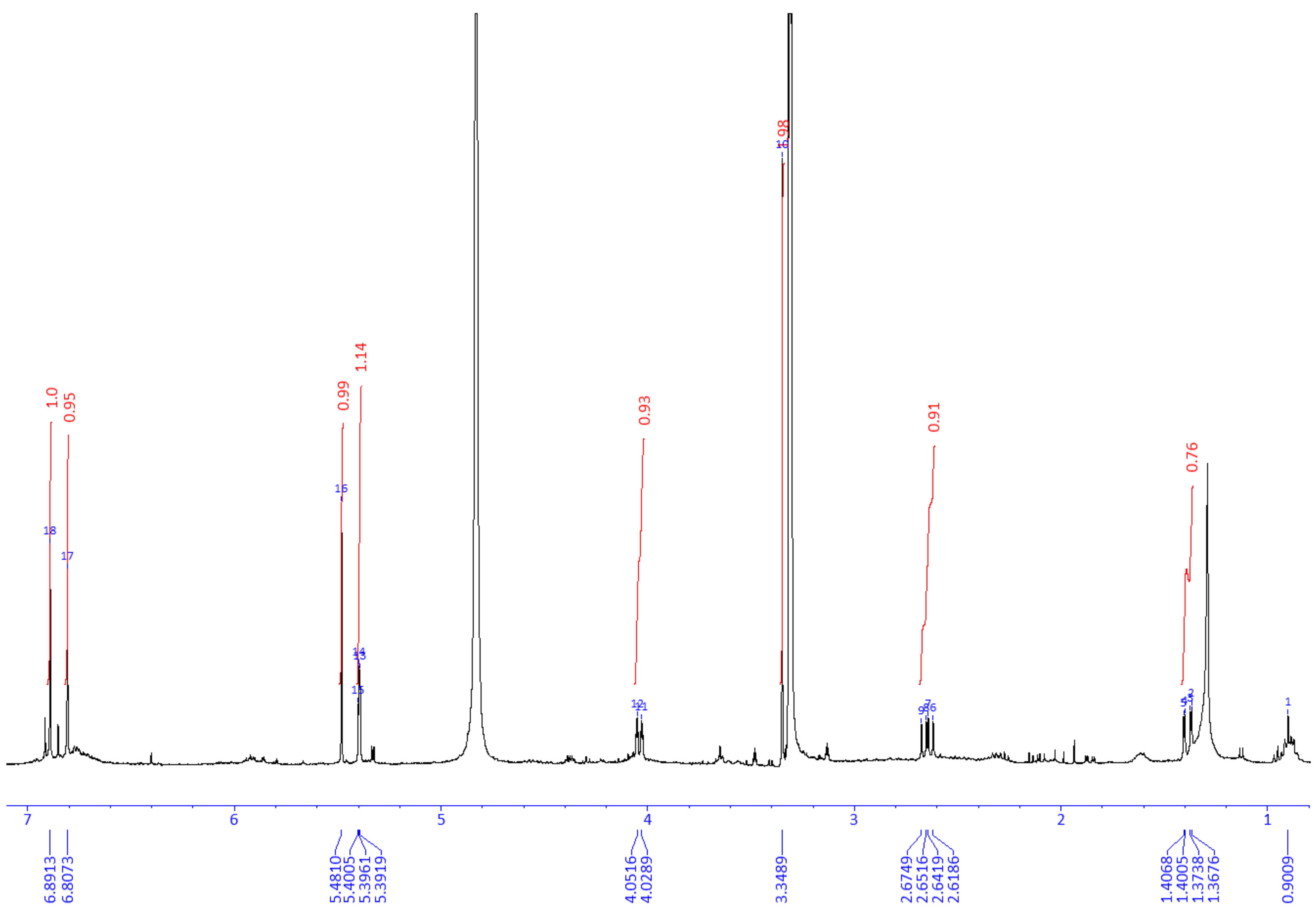

Fig. S3 ^{1}H NMR spectrum of peak 1 at 400MHz in CD_3OD

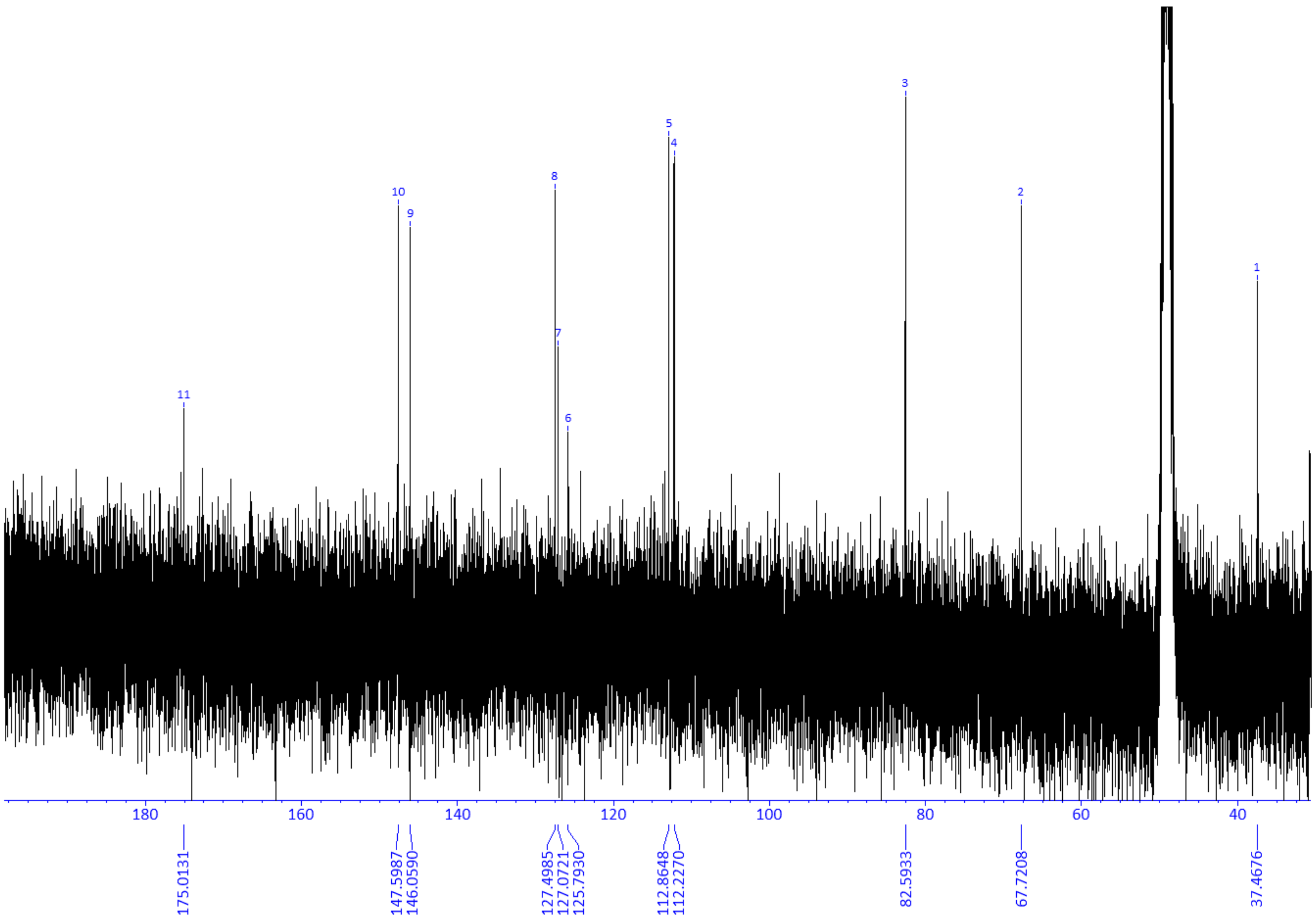

Fig. S4 ^{13}C NMR spectrum of peak 1 at 400MHz in CD_3OD

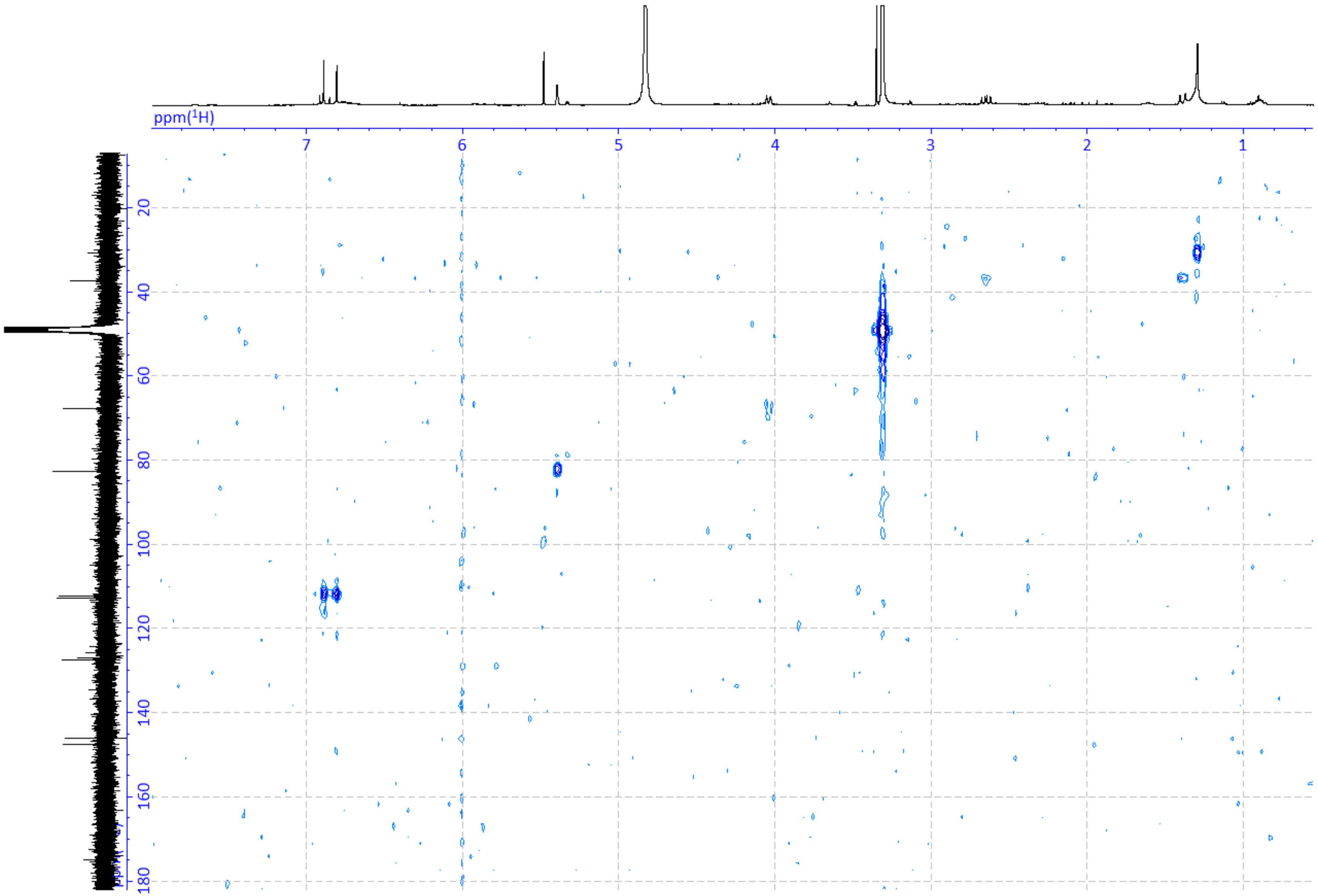

Fig. S5 gHMQC NMR spectrum of peak 1 at 400MHz in CD_3OD

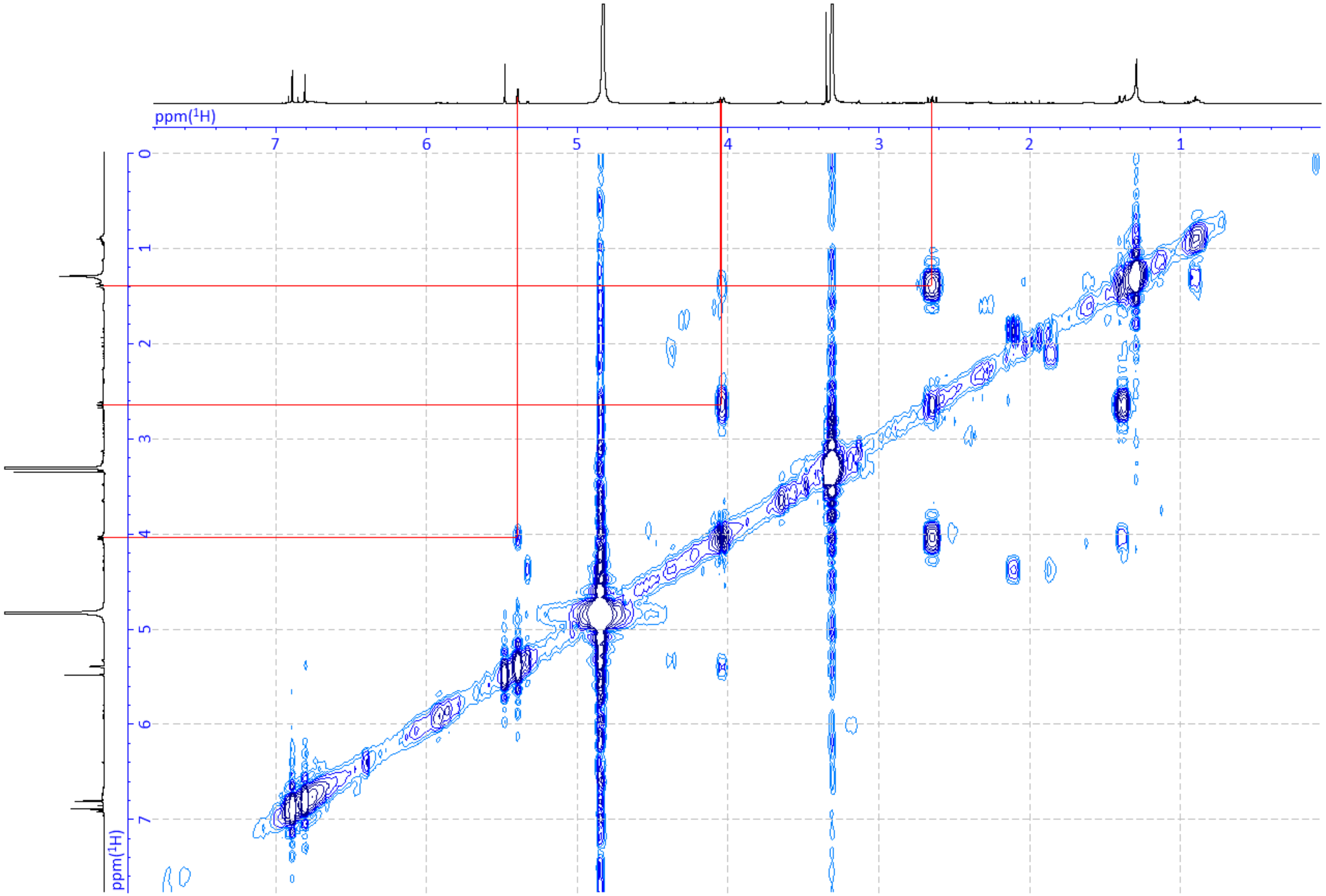

Fig. S6 gCOSY NMR spectrum of peak 1 at 400MHz in CD_3OD

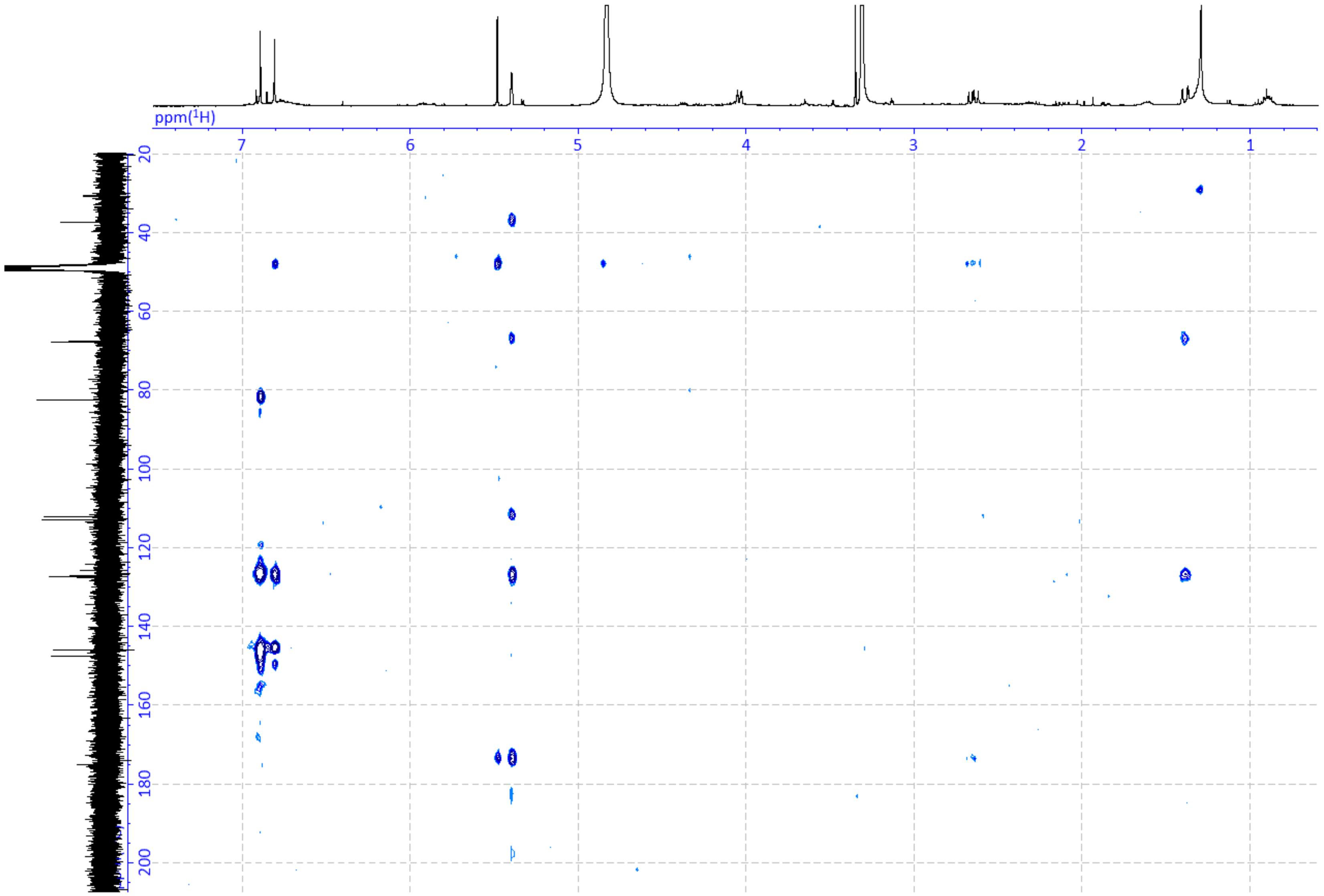

Fig. S7 gHMBC NMR spectrum of peak 1 at 400MHz in CD_3OD

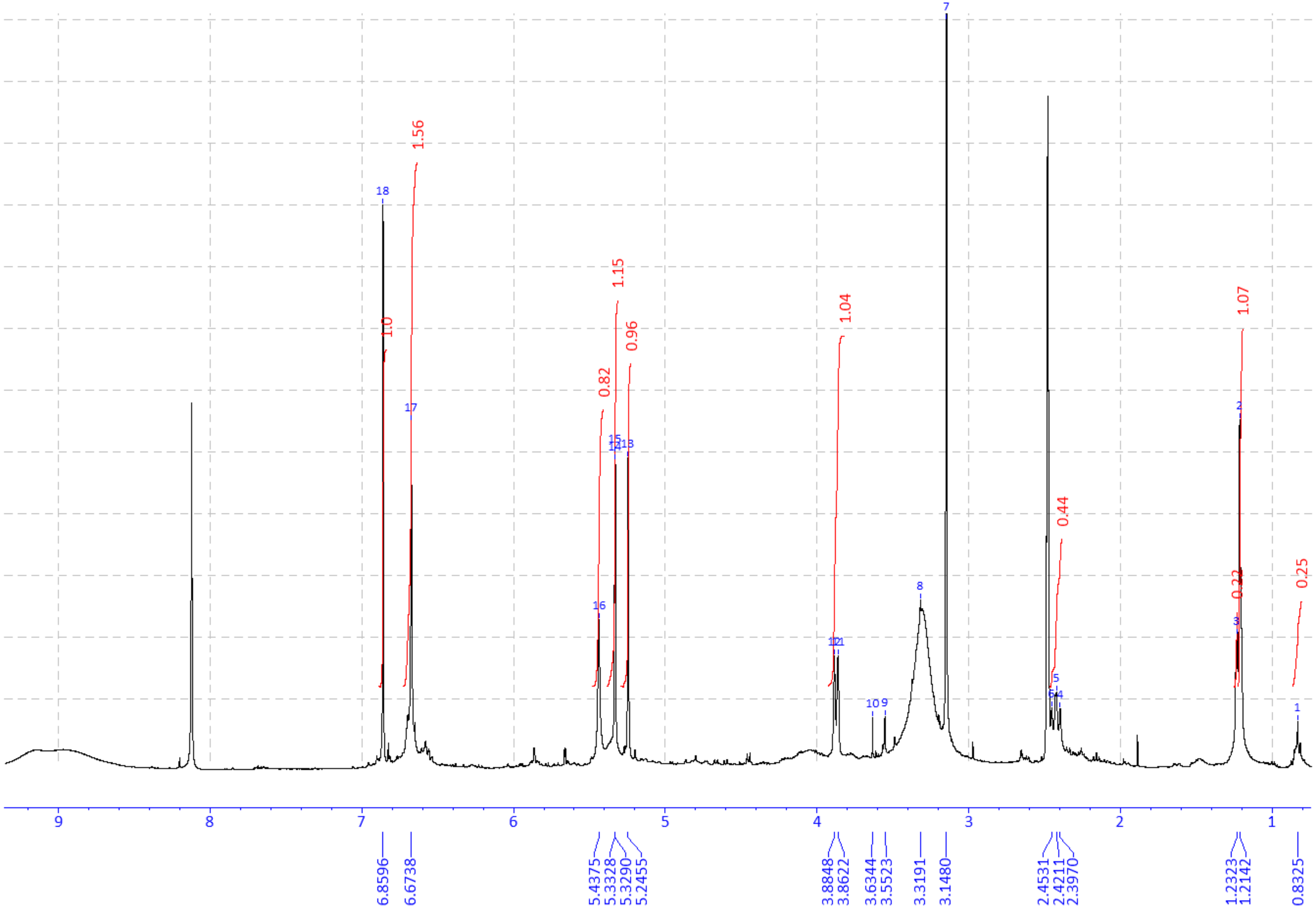

Fig. S8 ^{1}H NMR spectrum of peak 1 at 400MHz in DMSOd_6

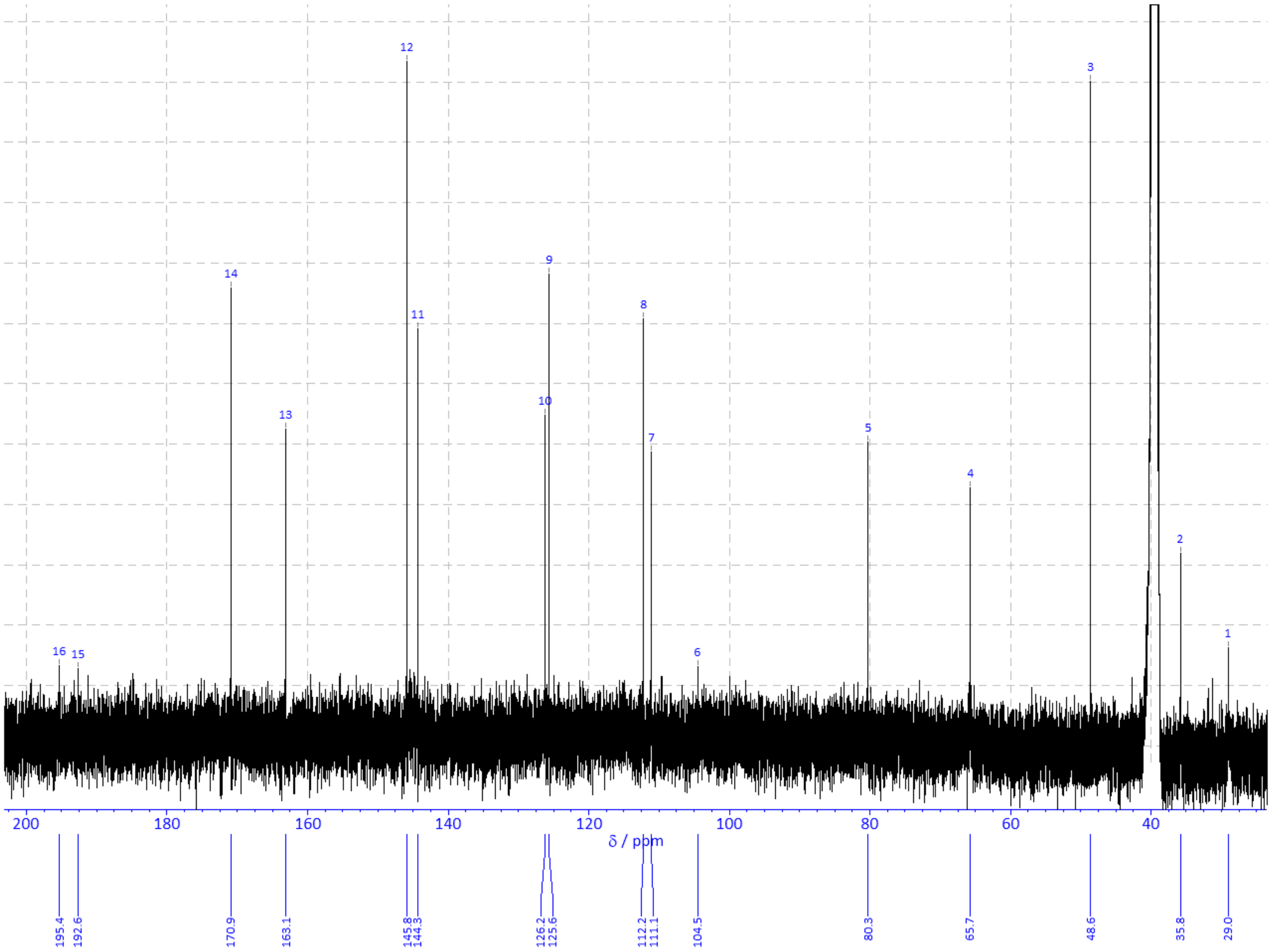

Fig. S9 ^{13}C NMR spectrum of peak 1 at 400MHz in DMSO-d_6

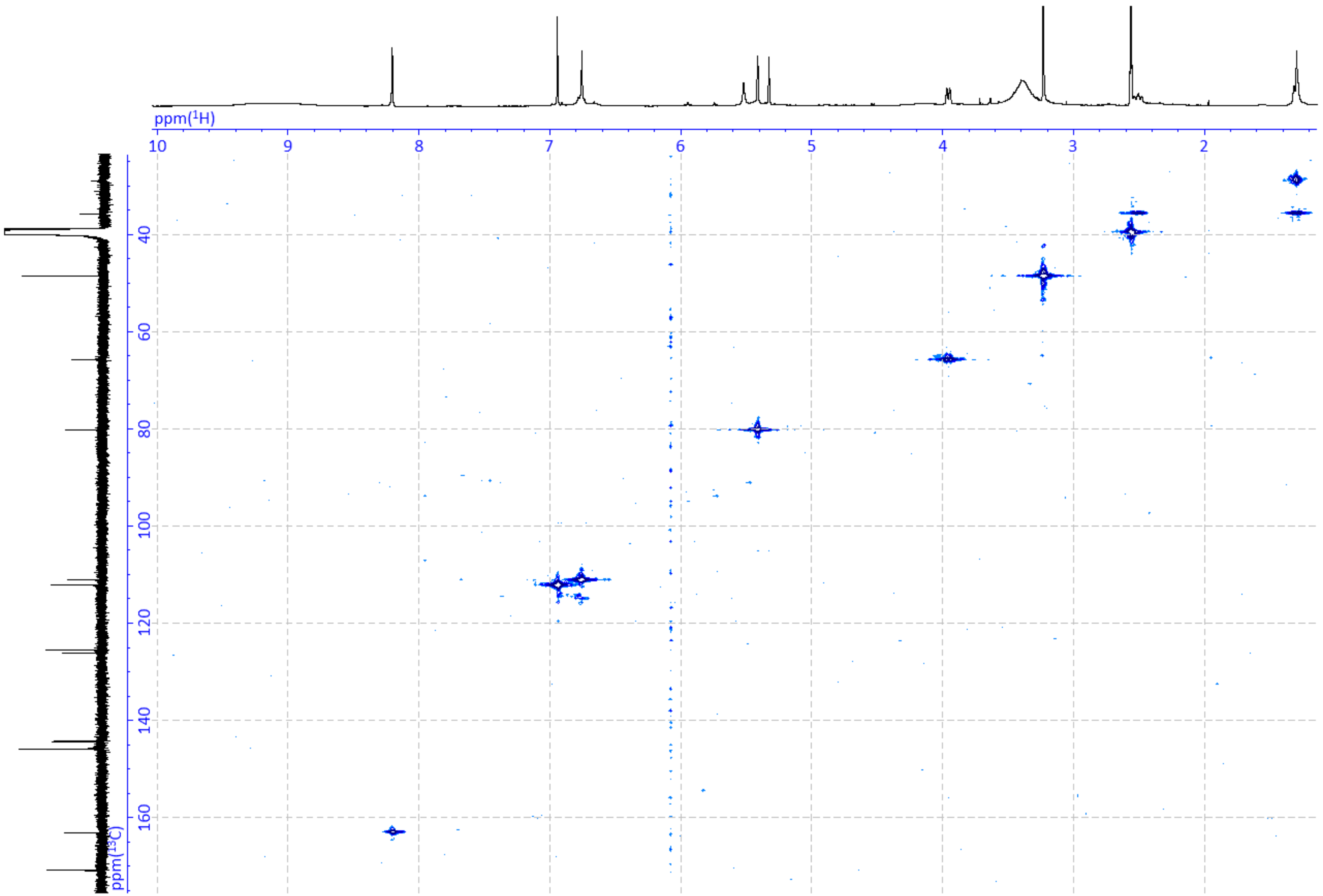

Fig. S10 gHMQC NMR spectrum of peak 1 at 400MHz in DMSO-d_6

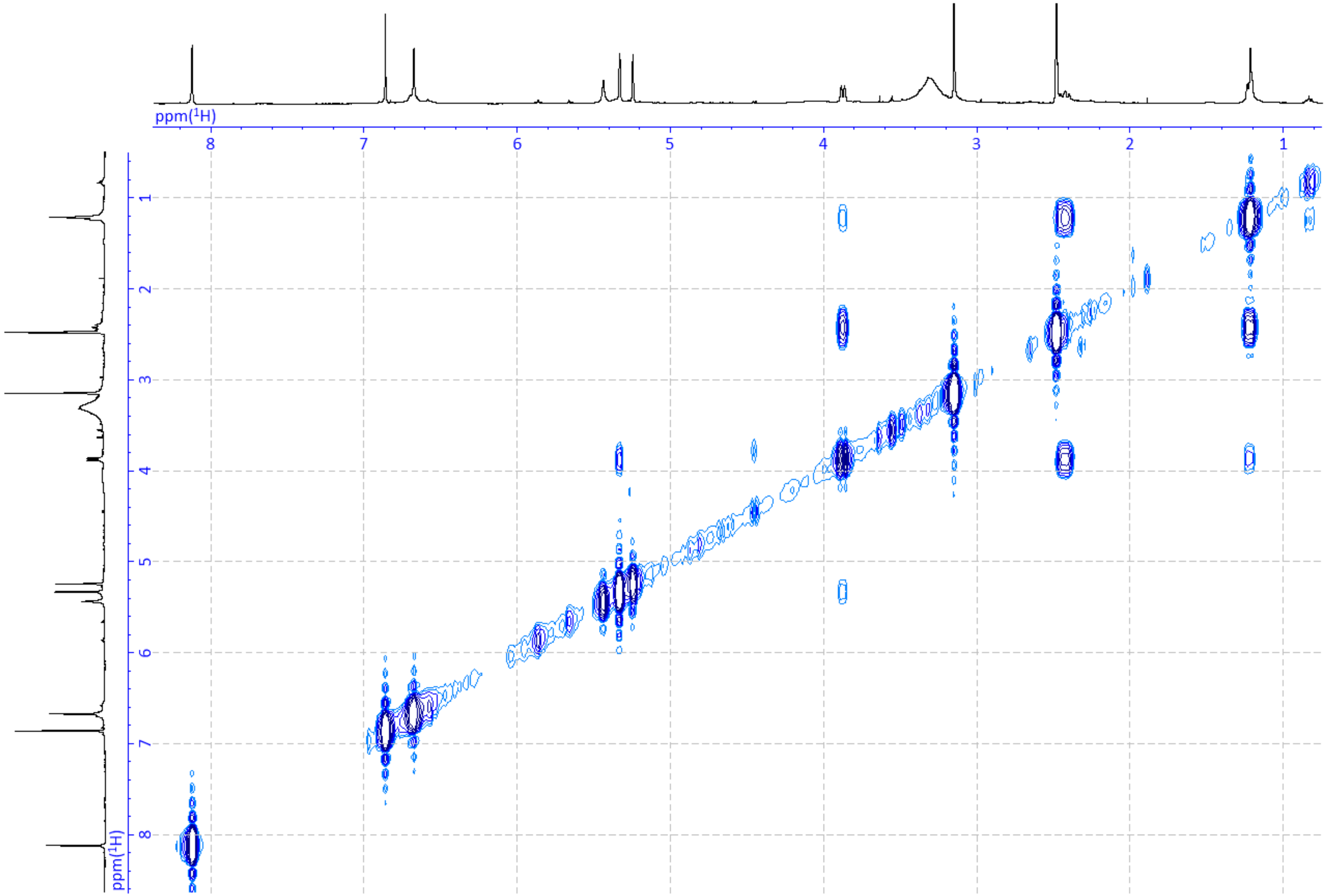

Fig. S11 gCOSY NMR spectrum of peak 1 at 400MHz in DMSO-d_6

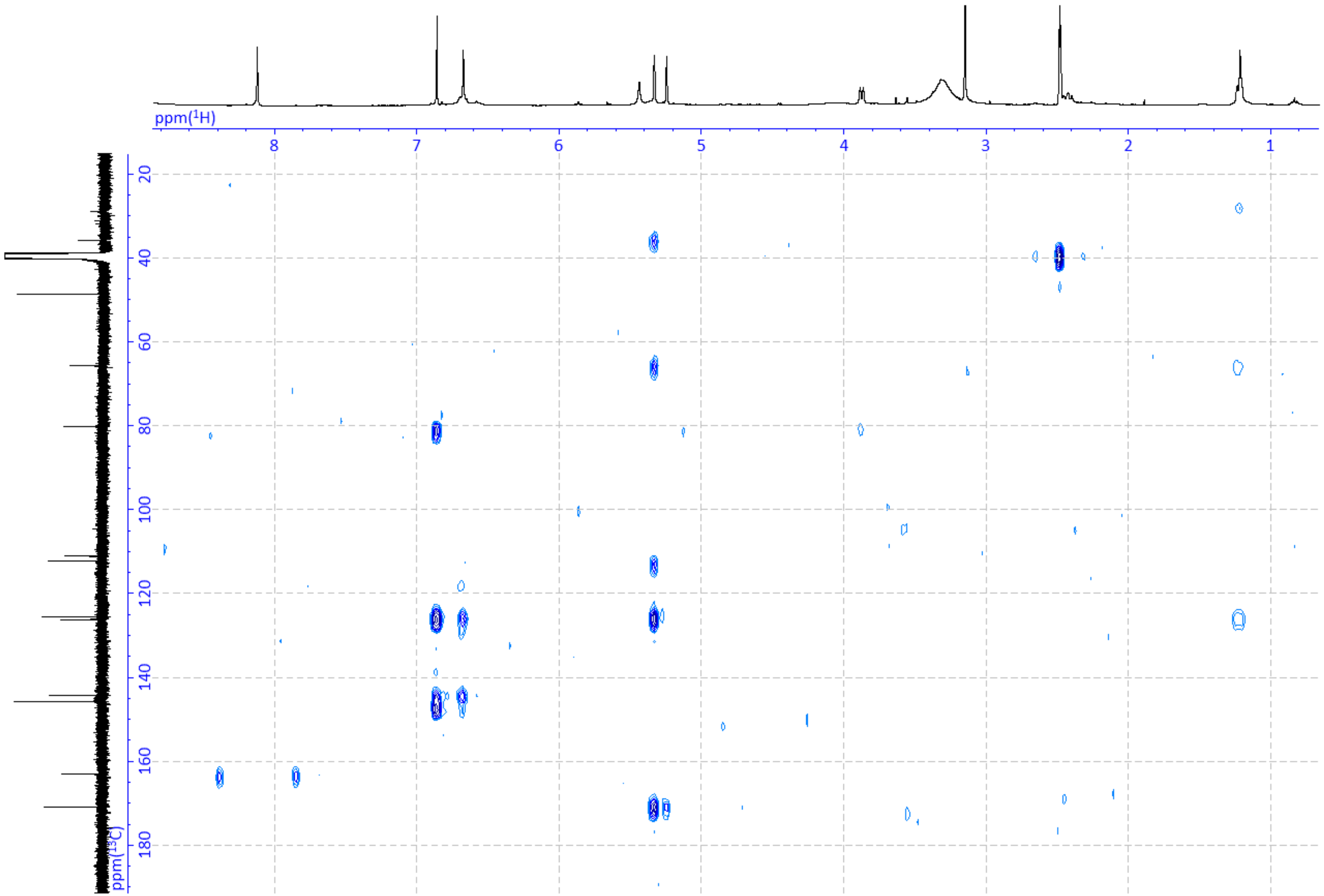

Fig. S12 gHMBC NMR spectrum of peak 1 at 400MHz in DMSO-d_6

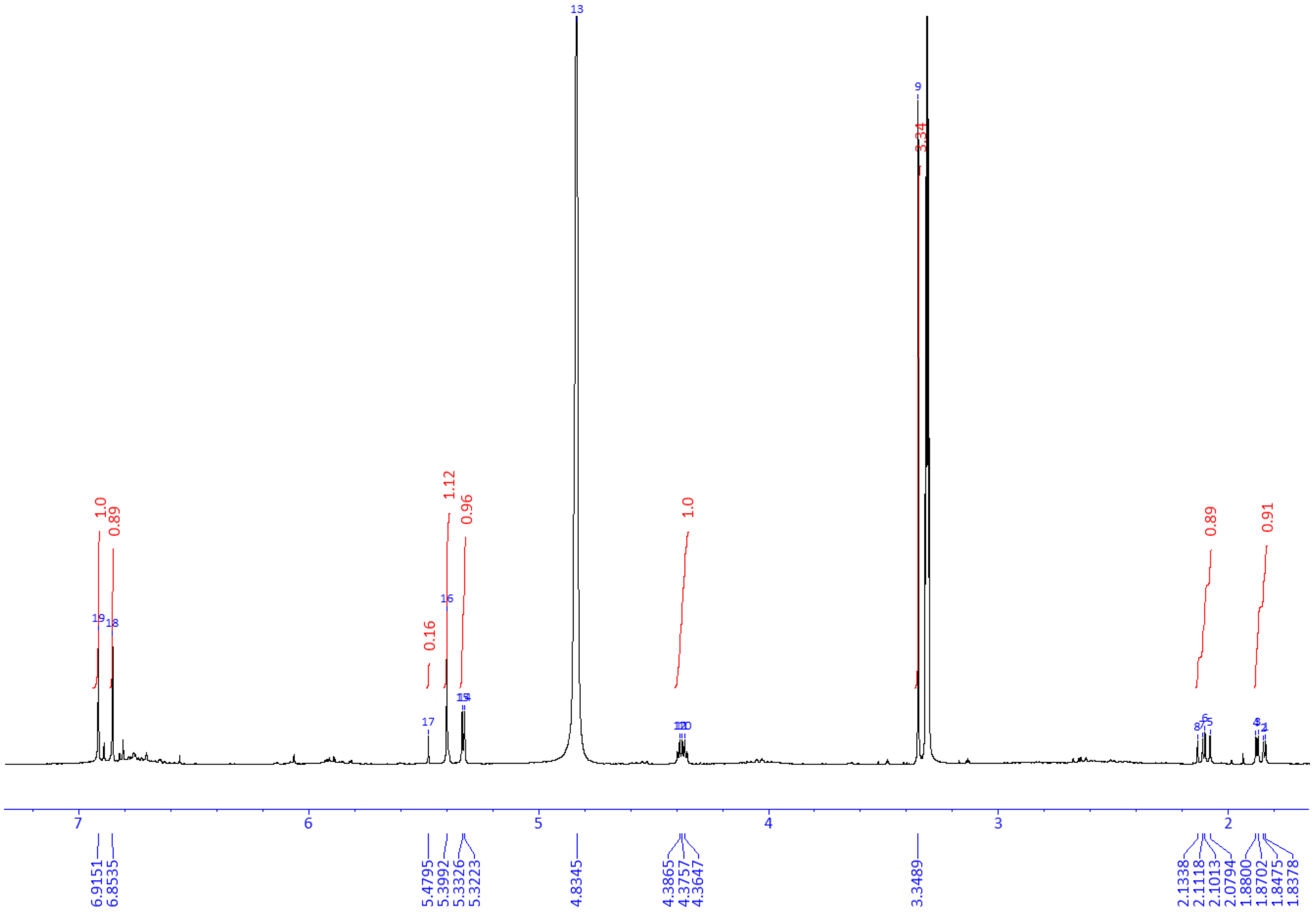

Fig. S13 ^{1}H NMR spectrum of peak 3 at 400MHz in CD_3OD

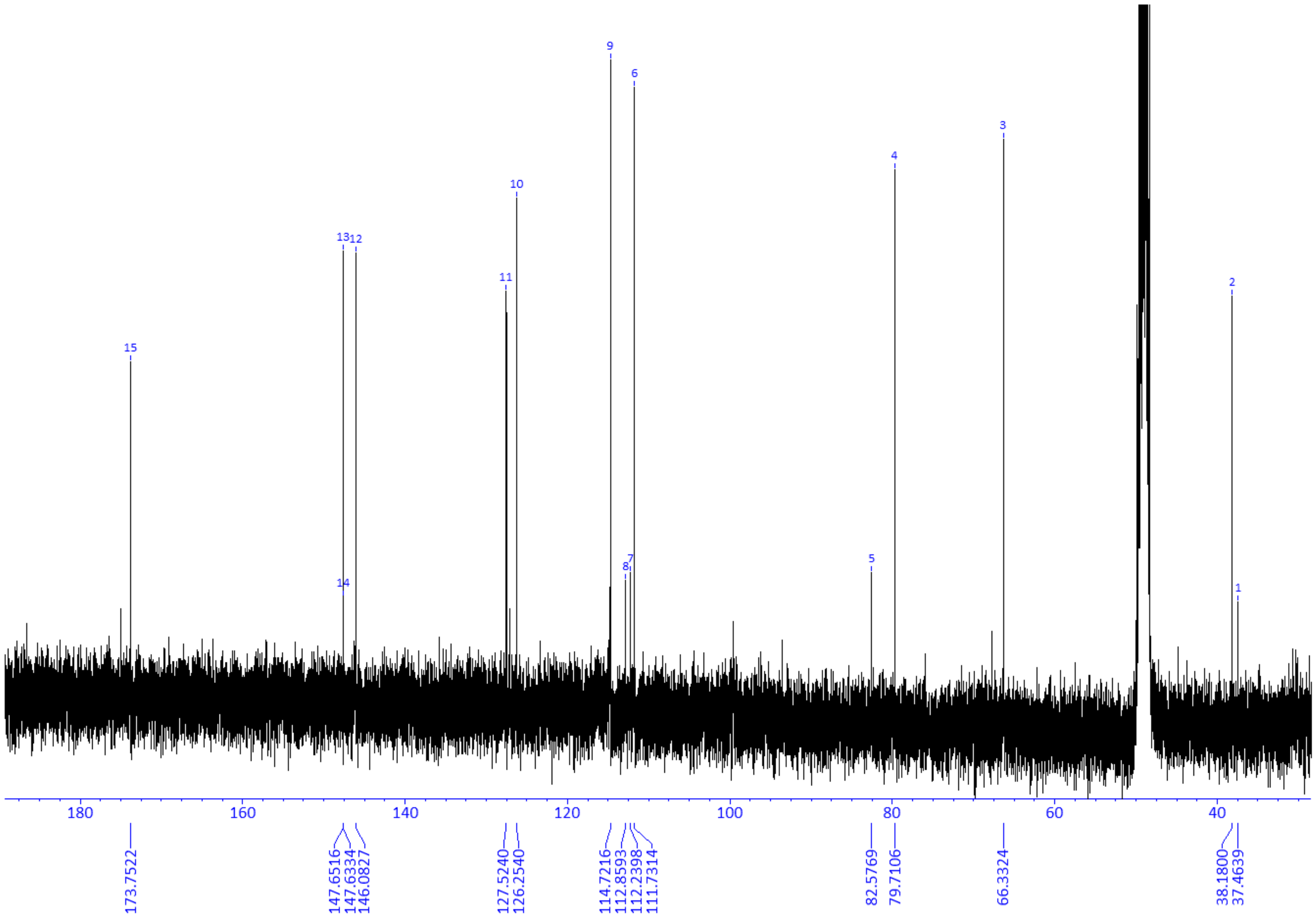

Fig. S14 ^{13}C NMR spectrum of peak 3 at 400MHz in CD_3OD

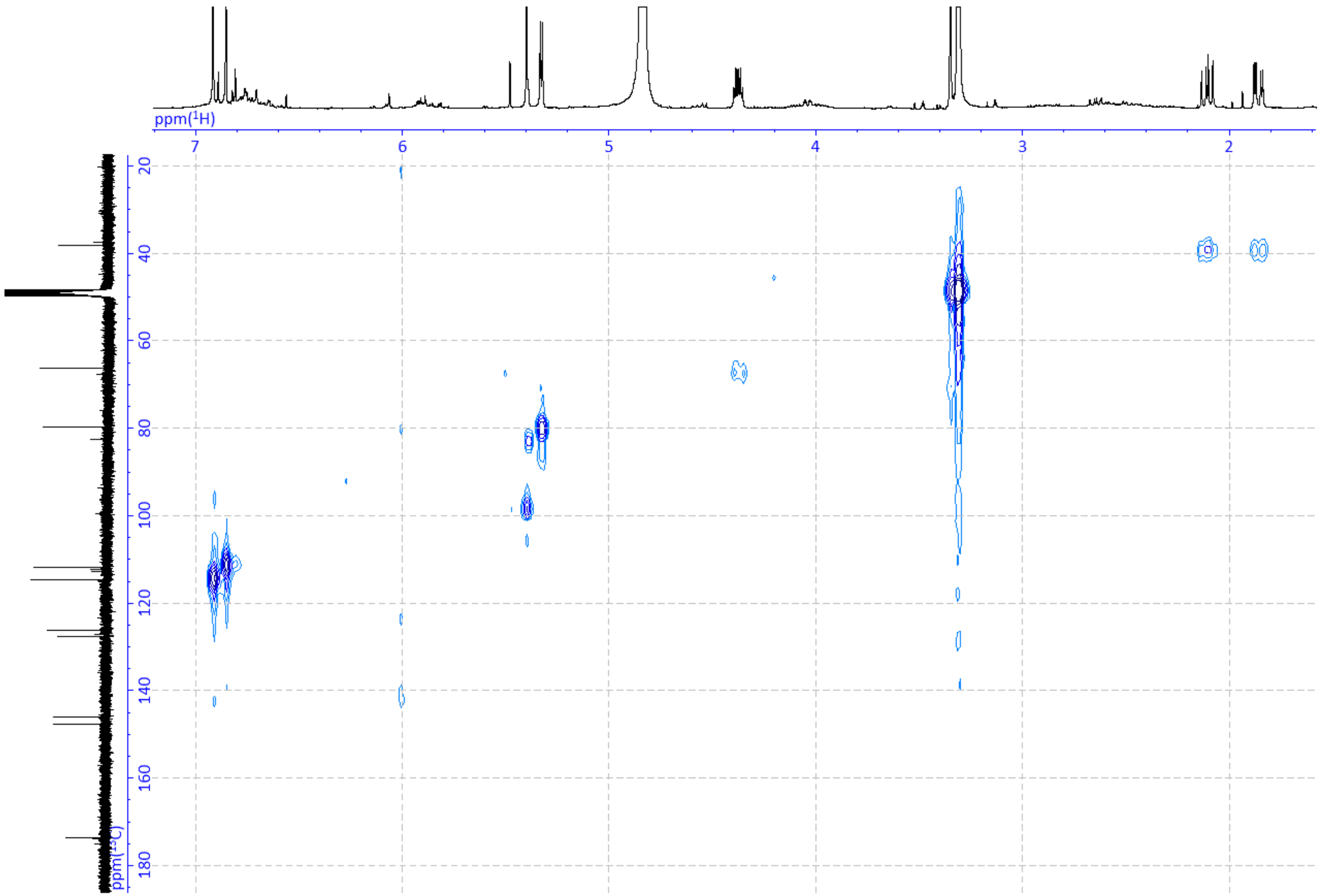

Fig. S15 gHMQC NMR spectrum of peak 3 at 400MHz in CD_3OD

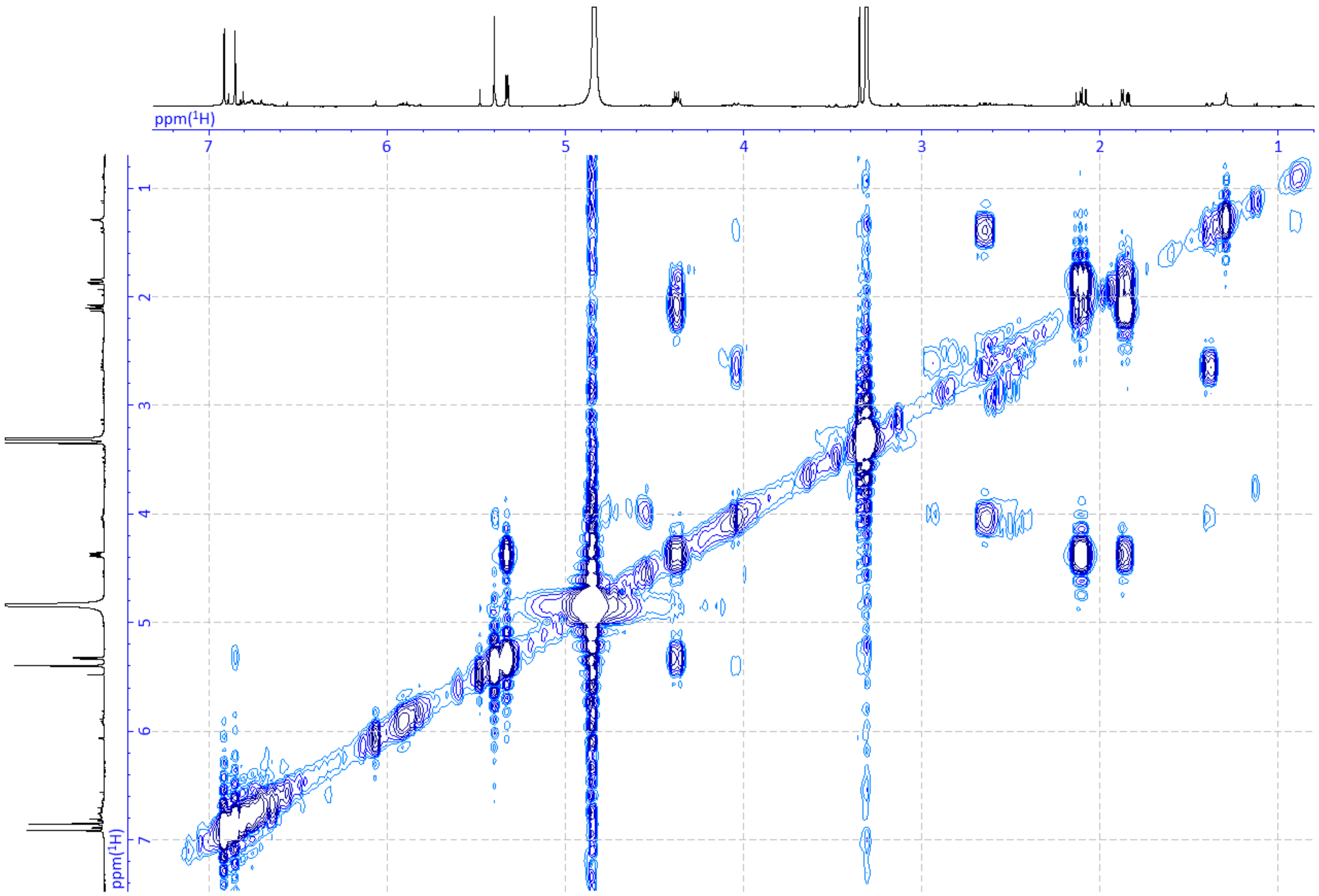

Fig. S16 gCOSY NMR spectrum of peak 3 at 400MHz in CD_3OD

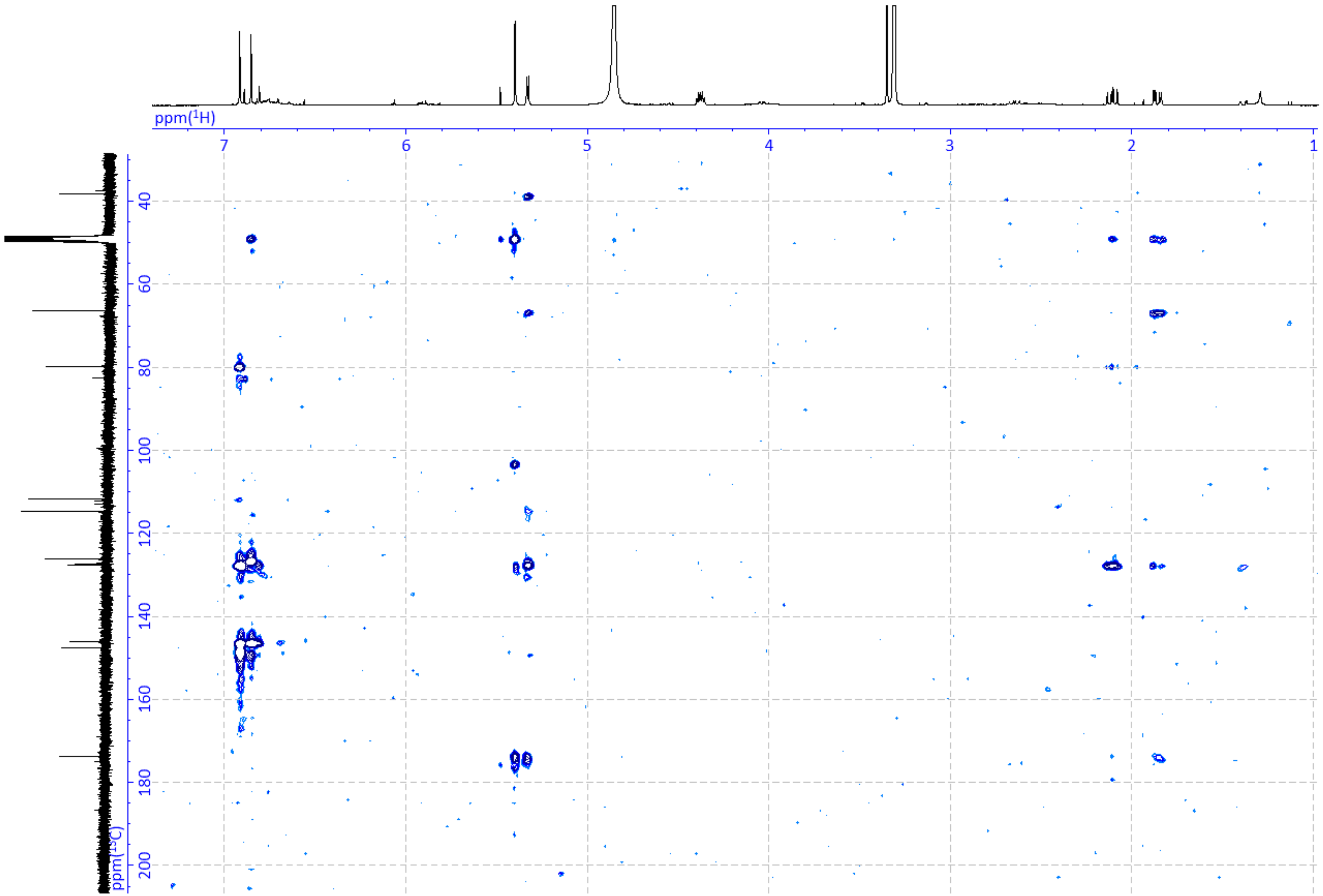

Fig. S17 gHMBC NMR spectrum of peak 3 at 400MHz in CD_3OD

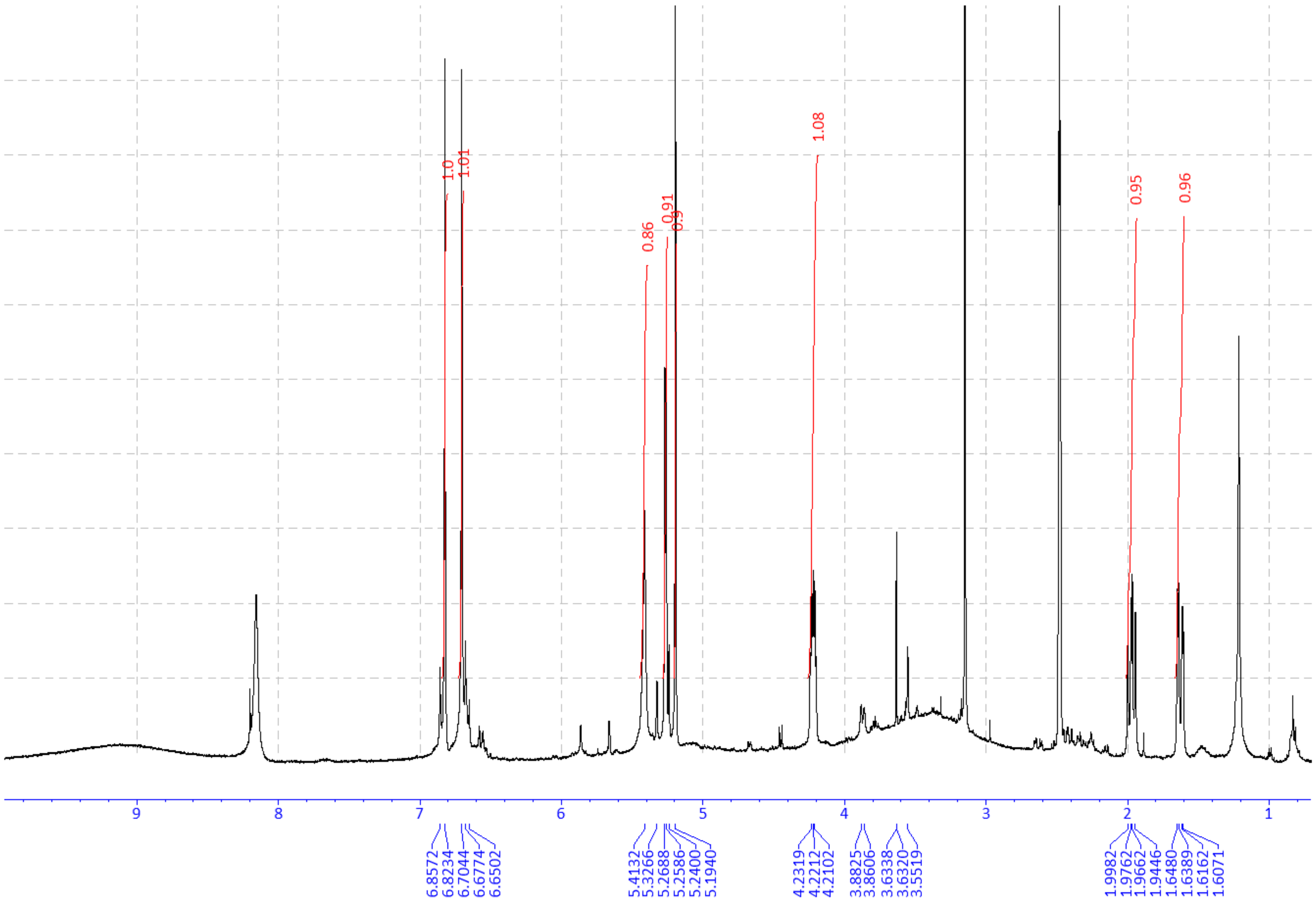

Fig. S18 ^{1}H NMR spectrum of peak 3 at 400MHz in DMSO-d_6

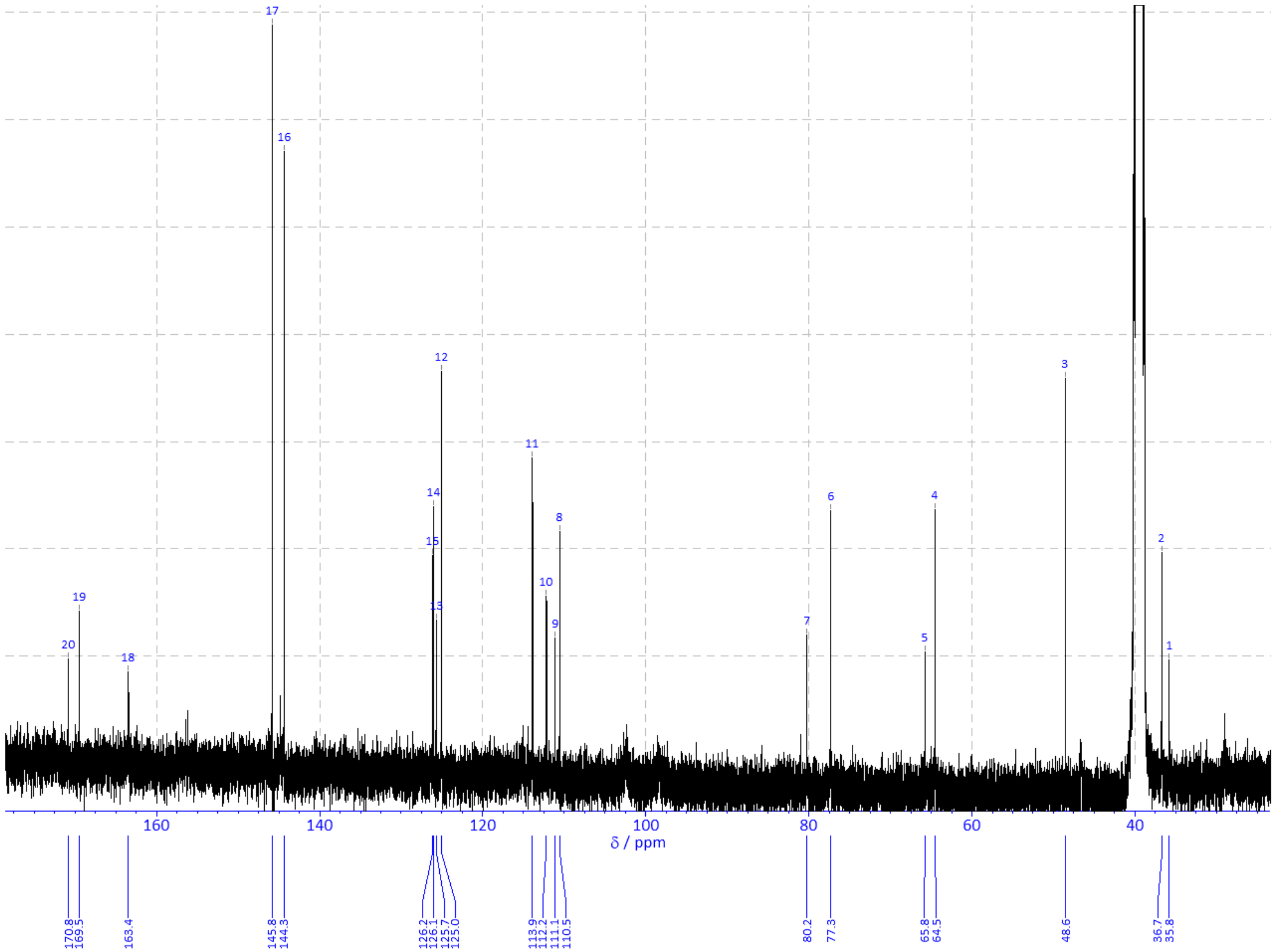

Fig. S19 ^{13}C NMR spectrum of peak 3 at 400MHz in DMSO-d_6

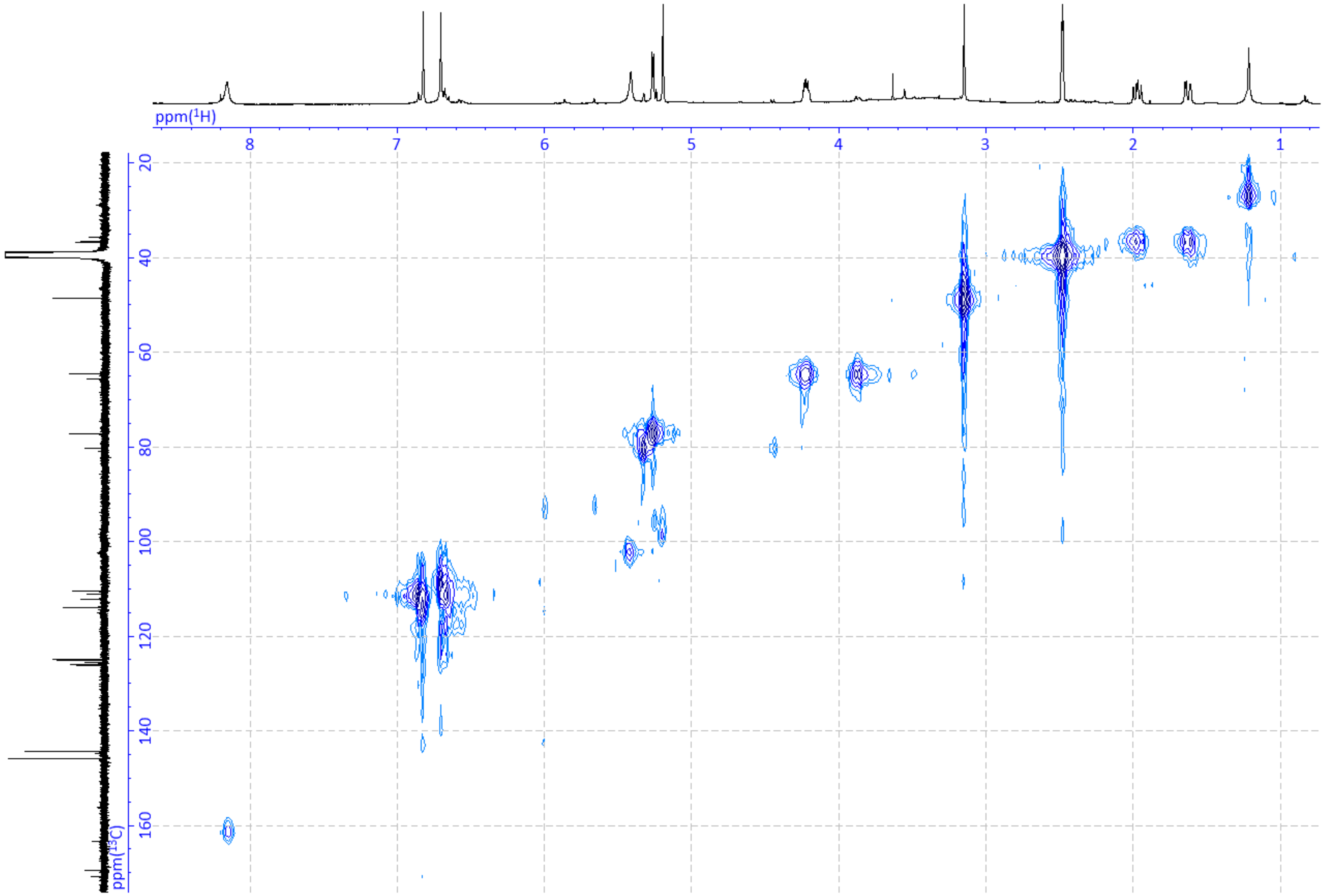

Fig. S20 gHMQC NMR spectrum of peak 3 at 400MHz in DMSO-d_6

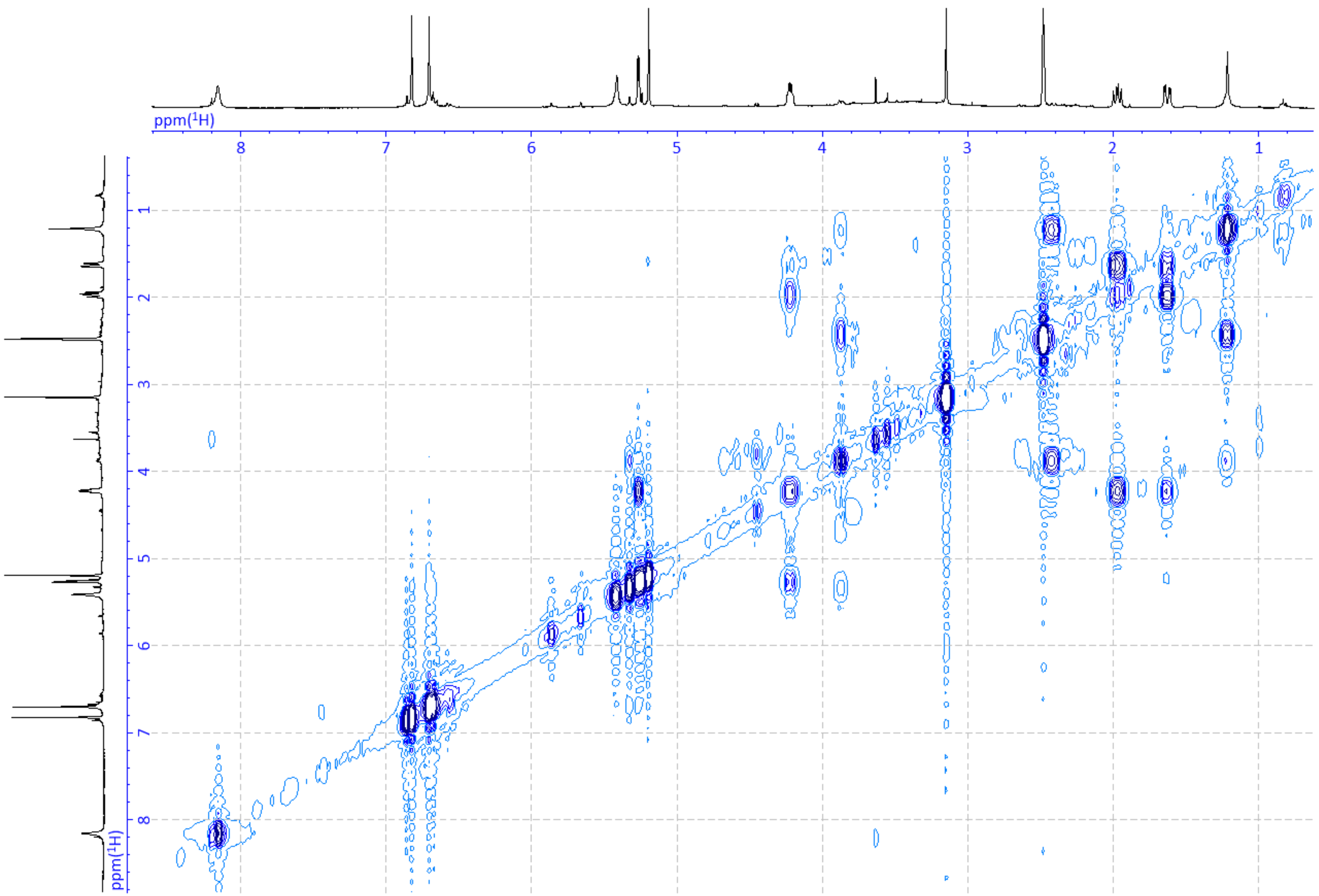

Fig. S21 gCOSY NMR spectrum of peak 3 at 400MHz in DMSO-d_6

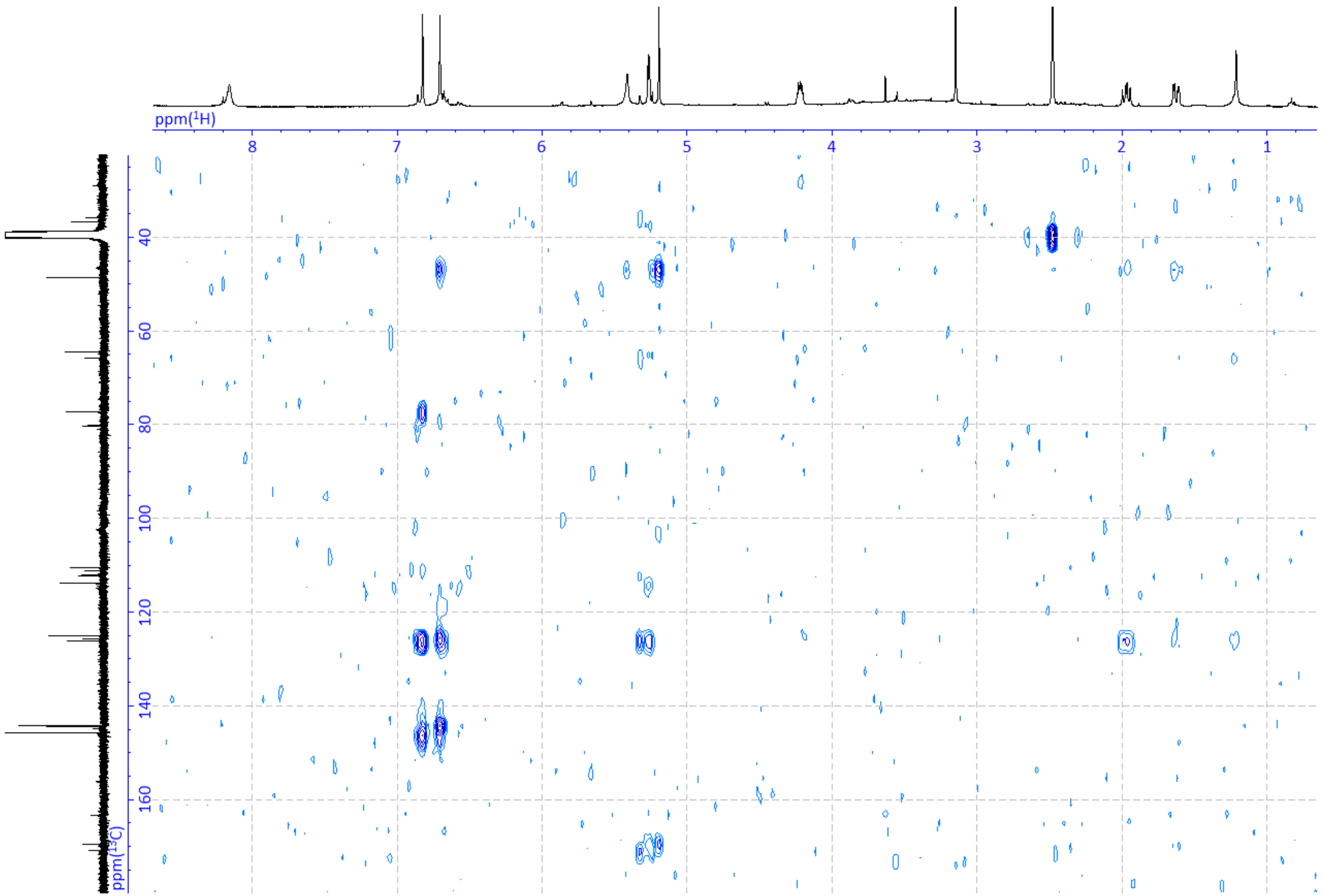

Fig. S22 gHMBC NMR spectrum of peak 3 at 400MHz in DMSO-d_6

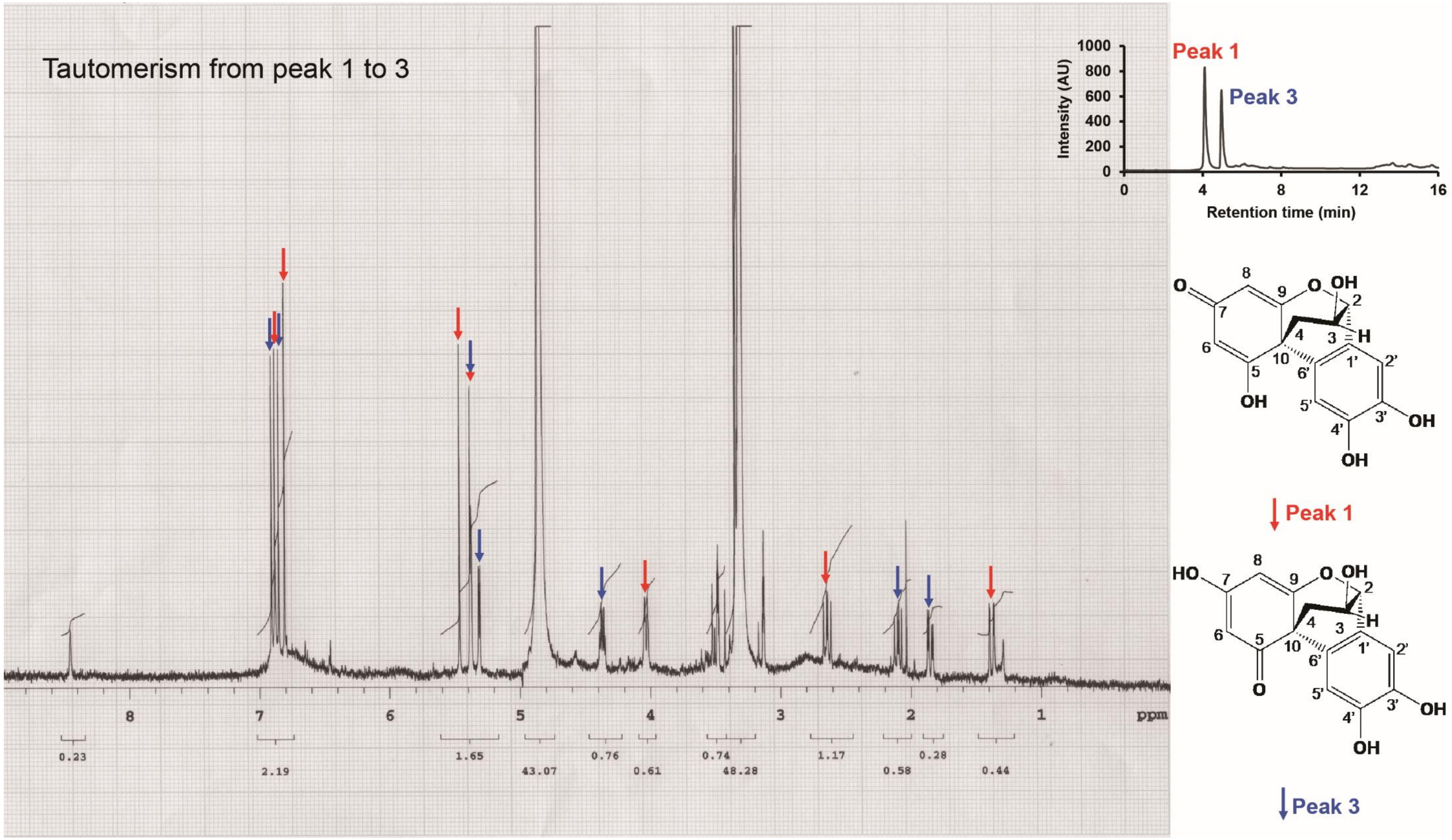

Fig. S23 ^{1}H NMR spectrum of peak 1 stored at 4°C for 4 weeks (tautomerism from peak 1 to 3).

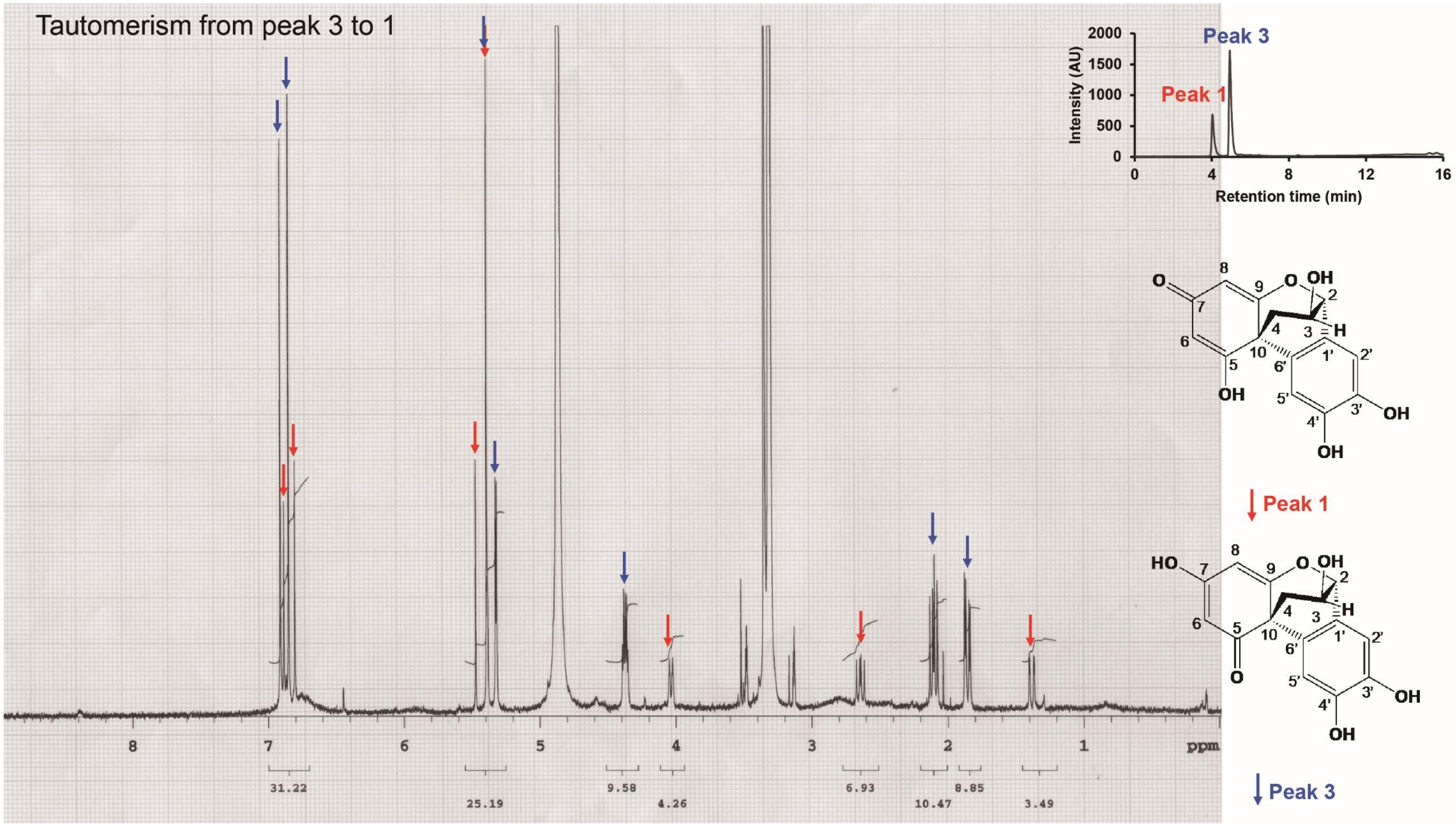

Fig. S24 ^{1}H NMR spectrum of peak 3 stored at 4 °C for 4 weeks (tautomerism from peak 3 to 1).

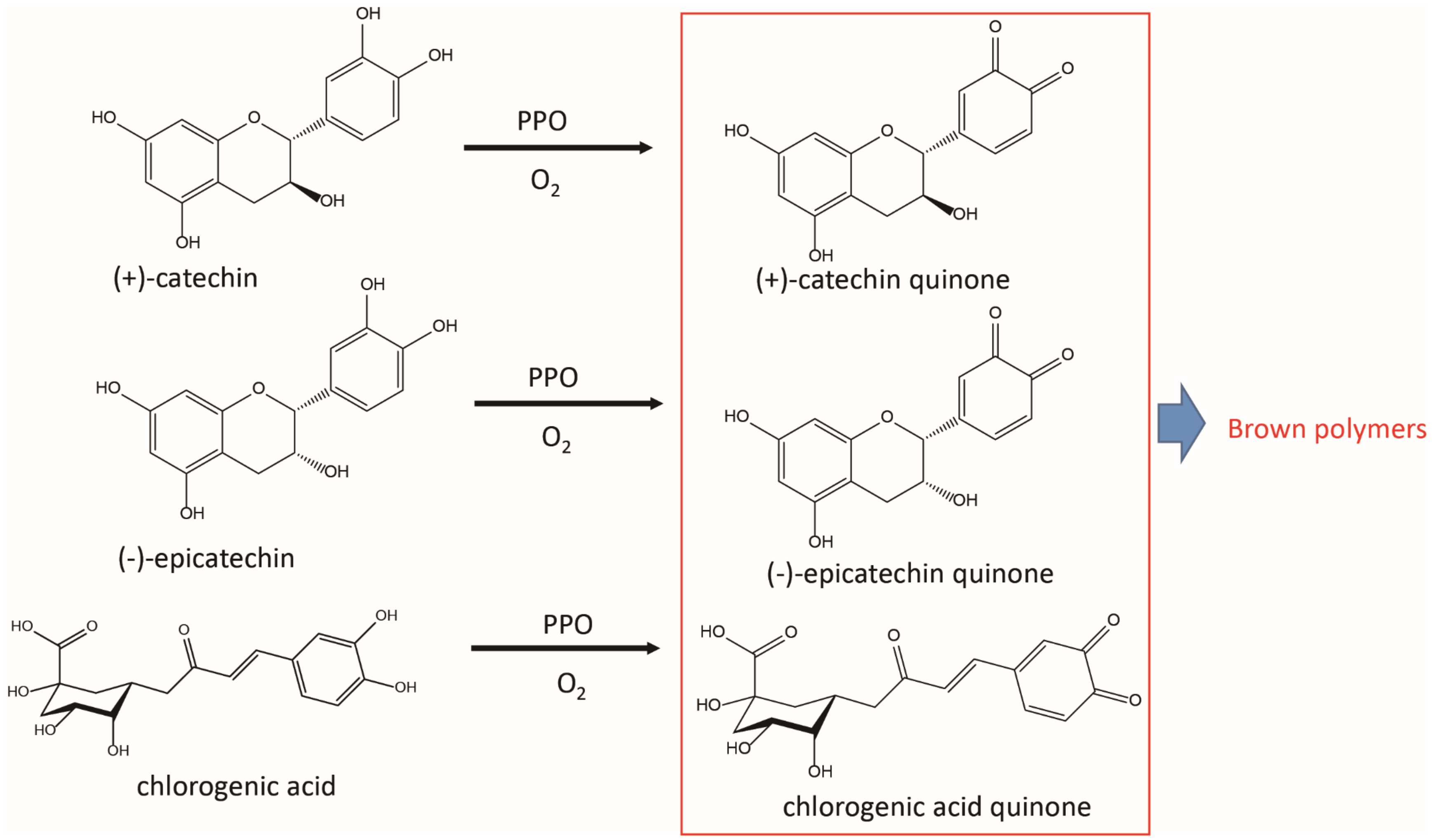

Fig. S25 Enzymatic browning of apple caused by polyphenol quinone polymers formed via polyphenols and polyphenol oxidase (PPO) in the presence of oxygen.

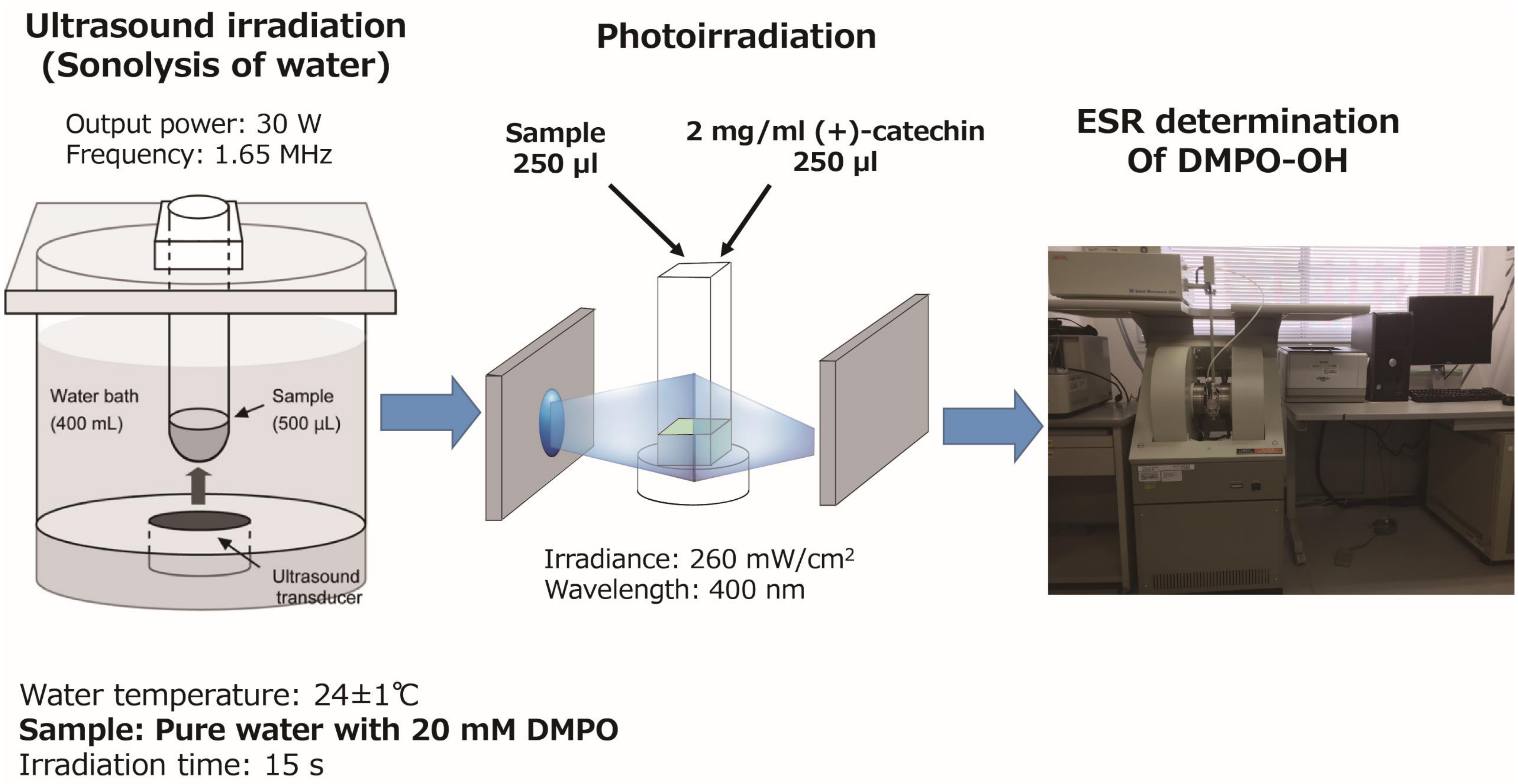

Fig. S26 Schematic illustration for examining the effect of photoirradiation of (+)-catechin on DMPO-OH generated by water sonolysis.
